# Characteristics and statistical analysis of university accidents in China from 2017 to 2021

**DOI:** 10.1016/j.heliyon.2023.e20616

**Published:** 2023-10-11

**Authors:** Guixiang Wu, Yanfei Yang, Chenglin Xu

**Affiliations:** School of Public Safety and Emergency Management, Kunming University of Science and Technology, Kunming, Yunnan, 650093, PR China

**Keywords:** University accidents, Logistic regression analysis, Influence strength value, Receiver operating characteristic curves

## Abstract

University accidents in China are frequent, and to find out the relationship pattern of factors influencing accidents, 248 university accidents occurring within 2017–2021 were studied using difference analysis (Independent-samples T-test, Mann-Whitney *U* test), logistic regression analysis, and diagnostic analysis of receiver operating characteristic curves. The results show: The variability in time, space, and qualifications was statistically significant (p < 0.05), and when the number of university safety policies ≥77 would significantly reduce the frequency of university accidents, with an influence strength value of 0.884 and a diagnostic accuracy of 79.8 %. In addition, the perpetrators, the time and the location of the accidents were usually undergraduate students, first semester of university, and economically developed and educationally rich provinces, respectively, with influence strength value and diagnostic accuracy of greater than 1 and 70%, respectively. Finally, specific suggestions are offered for the future prevention and reduction of accidents at the University based on the findings of the studies.

## 1 Introduction

China's National Bureau of Statistics (NBS) announced an increase in the number of students enrolled in universities from 30 million to 38 million for the period of 2017–2021 [1], and the large increase in the number of students enrolled in universities has led to a high number of university accidents. A number of university accidents at Fudan University, Beijing Jiaotong University, and Nanjing University of Aeronautics and Astronautics resulted in the deaths of 5 students. To prevent and lessen the frequency of accidents in universities, the General Office of the People's Republic of China's Ministry of Education published “Guidelines for Fire Safety in Universities and Colleges,” “Circular on Strengthening the Safety of Teaching Laboratories in Universities,” and “Circular on the Activities of the “Work Safety Month.” In response to the seriousness of the consequences of university accidents, it is therefore beneficial to prevent and reduce university accidents by conducting statistics and analyses of university accidents and exploring the underlying patterns of relationships.

Numerous researchers have undertaken different studies on university accidents, focusing on five areas: university safety culture, university fires, laboratory safety, university emergency preparedness, and university student suicides. In terms of university safety culture, Yang Fuqiang [2] used the ANP-SWOT model to analyze the internal and external environmental factors for building a university safety culture. Md. Khalid Hasan [3] and Yunhua Gong [4] conducted a questionnaire survey on university safety culture and discussed the factors related to university safety culture. In terms of university fires, Die Meng [5] used questionnaires to investigate the weaknesses in university students' firefighting knowledge. A Bayesian network structure model was created by Kaixin Yin [6] for studying the factors that cause university fires. Lu.Zhang [7] Building a university firefighting knowledge platform based on Unity3D. Yuanjun Wu [8] improved hybrid genetic algorithms to determine the best escape routes in university fires. In the area of university laboratory safety, Qian Li [9] used the Behavioural Safety Management model for the management methods of chemical laboratories. To improve the laboratory accident rescue plans, Yukun Gao [10] established a risk information collection and transmission system. Based on an analysis of 110 laboratory accidents, Mingqi Bai [11] offered the direction of control of the current situation and challenges of laboratory safety in Chinese universities. The application of process safety management principles to laboratory management is examined by Tomasz Olewski [12]. Inaam M. Nasrallah [13] conducted a risk factor assessment for science laboratories in Lebanese public universities. In terms of university emergency preparedness, Yu Liying [14] constructed a system dynamics (SD) model and an early warning management model based on SD and hyper network theory and explored the formation mechanism of a university public crisis. Christofer Skurka [15] conducted research on emergency preparedness in universities using a control group. Both Sharad Sharma and Shang Rong-Xue used virtual reality and ant colony optimization algorithms to study university emergency evacuation [16,17]. In order to investigate the variables influencing university student suicide, Wang Lingling [18] and Chen Jun [19] utilized stratified random sampling and questionnaires, respectively. Personalized feedback (PF) was utilized by Adam G. Horwitz [20] to investigate how much it affects college students' suicide ideation. Research on university accidents has so far concentrated on particular sorts of accidents, and no researchers have yet performed macro statistics and diagnostic analyses of university accidents, thus the field still needs to be further explored. In order to find patterns in the relationships between the factors influencing accidents, macro-statistical and diagnostic analyses of university accidents are of enormous practical importance.

## 2 Methodology

### 2.1 Data collection

China has not yet set up a special government agency to keep statistics and publish university accidents. This data mainly comes from authoritative media such as the official website of the Ministry of Emergency Management of the People's Republic of China, the official website of the National Bureau of Statistics of the People's Republic of China, People's Daily Online, Xinhua Daily Online and the official websites of the public security and fire departments of various regions, the China Emergency Education and Campus Safety Development Report (2017–2021), the China Statistical Yearbook (2017–2021), and the publicly available dissertation literature in the databases of CNKI, Wipro Chinese Journals, Wanfang, ElsevierSD, and so on. By checking the time and place of the accidents and removing duplicate university incidents, 248 accidents that occurred between 2017 and 2021 at universities were counted to confirm the accuracy of the data.

### 2.2 Analysis methods

Accident reports can provide valuable information, summarise lessons learned and explore trends in accident occurrence [21,22], and statistical investigation of accidents is an effective method [23,24] and can provide an important basis. To find out the relationship pattern of factors affecting university accidents and to verify the accuracy of the results, a single-factor analysis was used to screen the variability of time, space, and academic qualifications, a logistic regression model was constructed to obtain the influence strength values, and the results of the model were examined using the receiver operating characteristic curves (ROC).

#### 2.2.1 Logistic regression model

Logistic regression models are modeled with categorical variables as dependent variables and continuous or categorical variables as independent variables, which can measure the degree of influence of the independent variable on the dependent variable as well as explore risk factors [25,26].

The equation of the logistic regression model is as follows [27].

(1) $$\text{logit}\left(\rho_{x}\right)=\log\left(\frac{\rho_{x}}{1-\rho_{x}}\right)$$

(2) $$\text{logit}\left(\rho_{x}\right)=\beta_{0}+\beta_{1}\chi_{1}+\cdots {+\beta }_{n}\chi_{n}$$

(3) $$\ln\left(\frac{\rho_{x}}{1-\rho_{x}}\right)=\beta_{0}+\beta_{1}\chi_{1}+\cdots {+\beta }_{n}\chi_{n}$$

(4) $$\rho =\frac{e^{\left(\beta_{0}+\beta_{1}\chi_{1}+\cdots {+\beta }_{n}\chi_{n}\right)}}{1+e^{\left(\beta_{0}+\beta_{1}\chi_{1}+\cdots {+\beta }_{n}\chi_{n}\right)}}$$

(5) $$\ln\left({\text{OR}}_{n}\right)=\beta_{n}$$

(6) $${\text{OR}}_{n}=e^{\beta_{n}}$$

$\beta_{0}$ represents the constant term, ρ represents the probability of an accident, $\beta_{1}$、 $\beta_{2}\cdots \beta_{n}$ represent the logarithmic odds ratio (OR), ${\text{OR}}_{n}$ represents the odds ratio of the nth factor. In addition, odds ratios are influence strength values in this study OR are point estimates, and 95 % confidence intervals (95 % CI) are commonly used to account for sampling error.

#### 2.2.2 Model of ROC curves

The ROC curves rely on the area under the curve (AUC) to quantitatively evaluate the degree of excellence of the classifiers, covering all possible decision thresholds and thus allowing for the selection of the best thresholds for the model [[28], [29], [30]]. Thus, the ROC curves model provides an accurate judgment of the validity of the results of the logistic regression model.

1) Define classifiers and instances [31].
  ● True Positive (TP): The true result is a positive example and the prediction is a positive example.
  ● False Negative (FN): The true result is a positive example and the prediction is a negative example.
  ● True Negative (TN): The true result is a negative example and the prediction is a negative example.
  ● False Positive (FP): The true result is a negative example and the prediction is a positive example.

The true positive rate (TPR) and false positive rate (FPR) expressions are as follows [32]:

(7) $$\text{TPR}=\frac{\text{TP}}{\text{TP}+\text{FN}}$$

(8) $$\text{FPR}=\frac{\text{FP}}{\text{FP}+\text{TN}}=1-\text{Specificity}$$

**Fig. 1:**
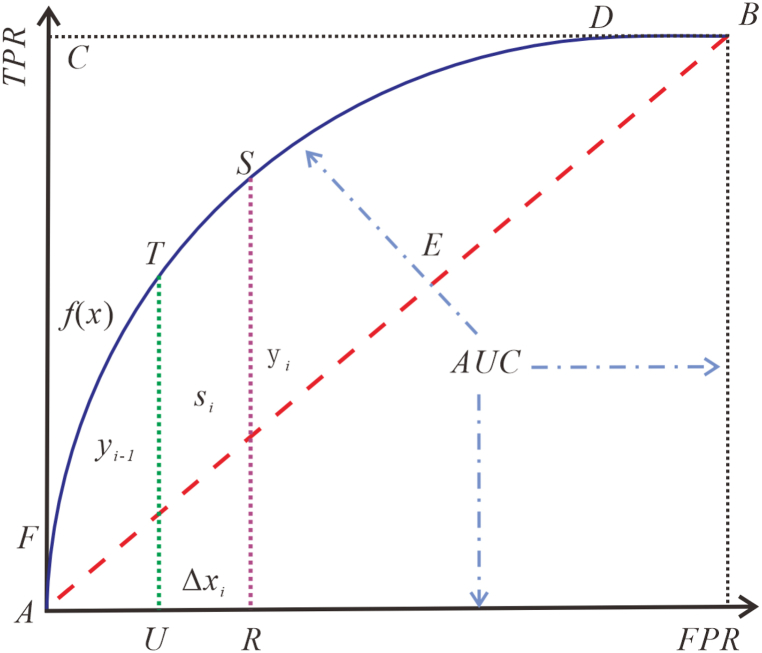
Schematic diagram of ROC curve coordinate system.

2) The ROC curves were established using the true positive rate (TPR) as the vertical coordinate and the false positive rate (FPR) as the horizontal coordinate as shown in Fig. 1 [33].

The expression for the area under the curve (AUC) is as follows [33]:

(9) $$\text{AUC}=\int_{e}^{f}f\left(\chi \right)d\chi =\sum_{i=1}^{n}S_{i}=\sum_{i=1}^{n}\frac{y_{i-1}+y_{i}}{2}{\Delta \chi }_{i}$$

e and f are the lower and upper limits of the horizontal coordinate FPR, respectively. $S_{i}$ is the area of the ith curved edge trapezoid of the ROC curves coordinate system. $y_{i-1}$, $y_{i}$, and ${\Delta \chi }_{i}$ are the upper base, lower base, and height of the curved trapezoid, respectively.

## 3 Characteristics of university accidents

**Fig. 2:**
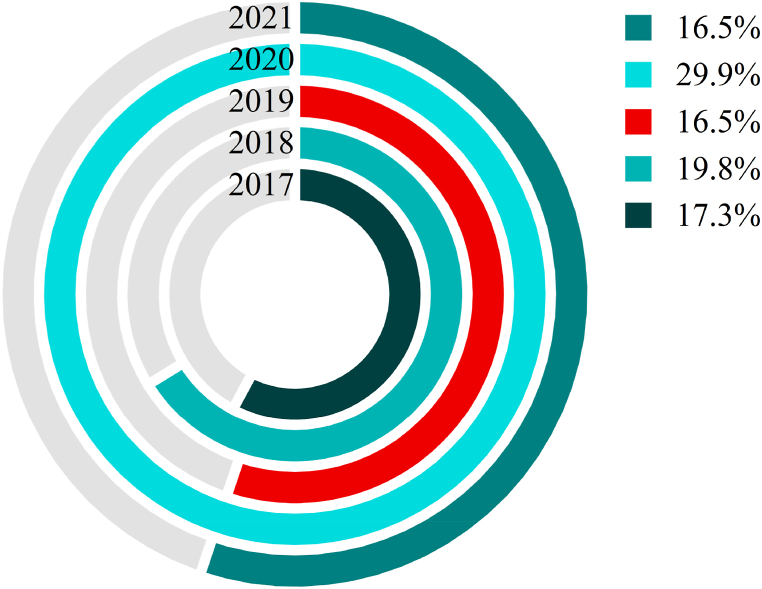
2017–2021 accident share chart.

**Fig. 3:**
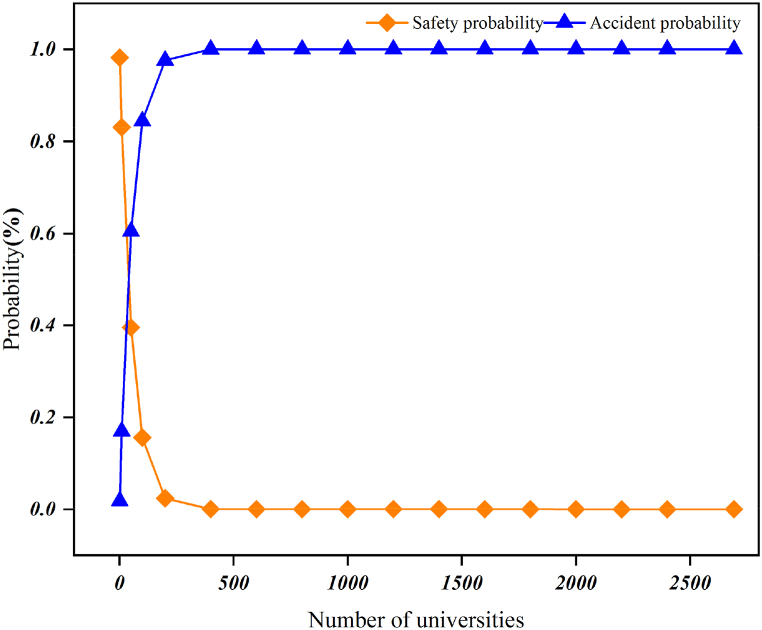
Probability cumulative chart.

Statistical analyses of university accidents can identify the potential relationship between accidents, and understand the status and trends of accidents, so as to be able to grasp the pattern of accidents from a macroscopic point of view, and can provide certain references and support for the prevention and reduction of university accidents. Fig. 2 shows that university accidents in 2017, 2018, 2019, and 2021 show a relatively stable trend, accounting for 17.3%, 19.8%, 16.5%, and 16.5% of the entire period, respectively, but the number of university accidents peaks in 2020, accounting for about 30% of the entire period. In the whole period, the average annual number of universities in China is about 2,692, the average annual number of accidents in universities is 49.6, the average annual probability of accidents is 1.84%, and the probability of safety is 98.16 %, and it can be seen from Fig. 3 that with the increase in the number of universities, the probability of accidents and the probability of safety show an opposite distribution. The probability of an accident and the probability of safety are approximately equal when the number of universities is 37, the probability of safety is about 15.61% and the probability of an accident is 84.39% when the number of universities reaches 100, and when the number of universities goes up to 1,000, the probability of safety is nearly 0 and the probability of accident is close to 100%. The number of universities in 2021 is 2738 [1], much larger than 1000. Therefore, university accidents have become a serious problem and the government, universities, and families must pay great attention to university accidents.

### 3.1 Characteristics of the time distribution

The temporal distribution of accidents is mainly in terms of year and month. Reveal the trend of accidents, and the characteristics of the time period, discuss the reasons for the occurrence of the time pattern of accidents, and be able to provide a basis for the determination of the frequency and intensity of monitoring by the management [34]. The number of times the university accidents was counted according to the year and month they occurred and the results are shown in Table 1.

**Table 1:** Distribution of accidents in different years, months and qualifications.

Year	Fire mishaps	Laboratory accidents	Unintentional mishaps	Suicide and homicide accidents	Number of policies	Injuries	Deaths
2017	20	2	6	15	80	2	22
2018	29	2	7	11	87	23	24
2019	25	1	4	11	82	6	21
2020	27	1	13	33	54	8	43
2021	21	4	8	8	83	13	18

**Month**	**Fire mishaps**	**Laboratory accidents**	**Unintentional mishaps**	**Suicide and homicide accidents**
Jan.	4	0	4	3
Feb.	3	1	1	3
Mar.	9	3	1	8
Apr.	7	0	3	6
May.	12	0	2	4
Jun.	6	0	1	7
Jul.	3	2	0	4
Aug.	5	0	0	4
Sep.	15	0	7	9
Oct.	19	1	2	15
Nov.	18	1	9	9
Dec.	21	2	8	6
**qualifications**	**Fire mishaps**	**Laboratory accidents**	**Unintentional mishaps**	**Suicide and homicide accidents**
Undergraduate (accident rate)	68 (86 %)	2 (20 %)	29 (81 %)	54 (68 %)
Postgraduates (accident rate)	11 (14 %)	8 (80 %)	7 (19 %)	25 (32 %)

From Table 1, we get the number of accidents in the university for the years 2017–2021 as 43, 49, 41, 74, 41, the number of fatal students as 22, 24, 21, 43, 18, and the number of injured students as 2, 23, 6, 8, 13, and the number of fatal students for the whole period as 128, and the number of injured students as 52. The frequency and percentage of fire mishaps, laboratory accidents, unintentional mishaps, suicide and homicide accidents were 122, 10, 38, 78; 49.19%, 4.03%, 15.32%, 31.46%, and the number of casualties and casualty rates were 30, 29, 37, 84; 16.67%, 16.11%, 20.55%, 46.67%, and the total accidental casualty rate was 72.58%. The 24 sudden deaths accounted for 63.16% of Unintentional mishaps, with suicide and homicide accidents accounting for nearly 100% of the death rate. Among them, the success rate of Automated External Defibrillator (AED) resuscitation for sudden death was 74 % in less than 3 min, and 49 % in more than 3 min [35], and only 2 students were successfully treated by AED in the whole period. The number of university accidents and fatalities peaked at 74 and 43 in 2020, combined with the number of policies on university safety from 2017 to 2020 in the table at 80, 87, 82, 54, and 83, respectively, with the number of policies in 2020 being the lowest for the entire period, suggesting that university accidents increased significantly as policies on university safety decreased, implying that a negative correlation between the two may have existed.

Table 1 shows that during the whole period, there were 69 accidents in March–June, 142 accidents in September–December, and 37 accidents during the summer and winter holidays, which accounted for 27.82%, 57.26%, and 14.92% of the accidents in the university for the whole year, respectively. The accidents show the phenomenon of the “autumn semester”, which is consistent with the phenomenon of the “first semester” proposed by Xiaofeng Hu [36]. In addition, the number of accidents is also higher in the “spring semester” than in the summer and winter holidays, which means that the number of accidents at the University may be affected by the “spring semester”. There is a significant increase in the number of university accidents in both periods, further suggesting that the “spring and autumn semesters” should be taken into account as a factor in preventing university accidents.

### 3.2 Provincial location distribution

**Fig. 4:**
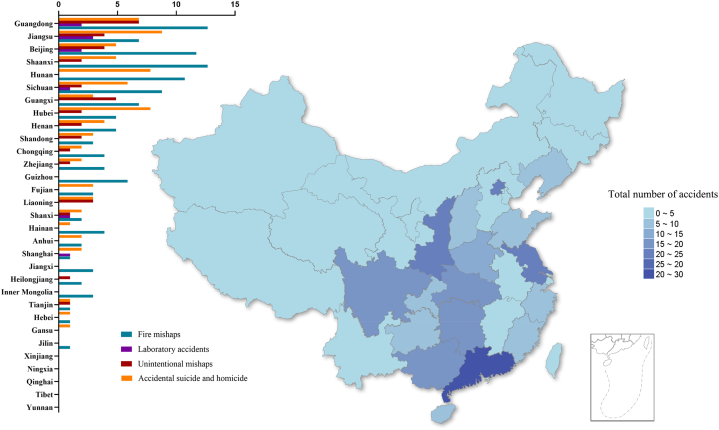
Distribution of accidents in different provinces.

**Fig. 5:**
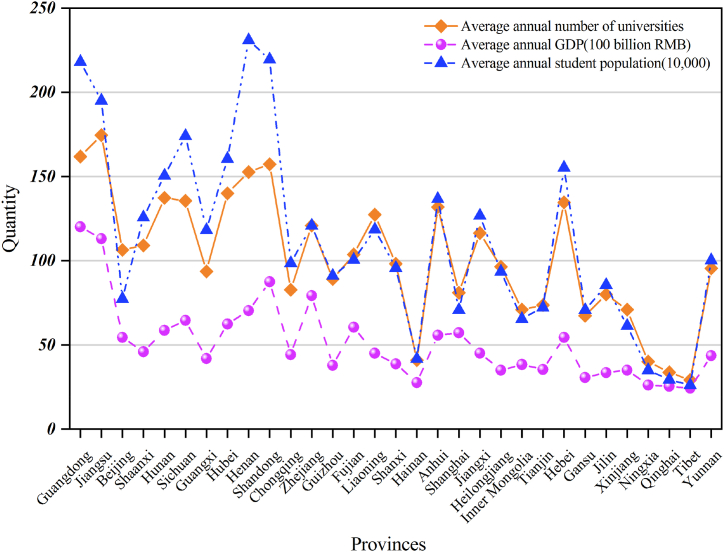
Data chart of different provinces.

Statistical data on university accidents, average annual Gross Domestic Product (GDP) (100 billion RMB), average annual student population (10,000), and average annual number of universities in 31 provinces, autonomous regions, and municipalities directly under the central government of China (excluding Taiwan, Hong Kong, and Macao) are shown in Fig. 4, Fig. 5.

The relatively economically developed regions in the figure, such as Beijing, Jiangsu, Hunan, Guangdong, Sichuan, and Shaanxi Provinces, have an average annual GDP (100 billion RMB) of 34.93, 99.38, 39.53, 107.02, 45.90 and 25.40, accounting for 36.11 % of China's average annual GDP. The average annual student population (10,000) in school is 60.14, 189.55, 140.55, 214.87, 166.56, and 113.32, accounting for 29.14 % of the average annual student population in schools in China. The average annual number of universities is 92, 167, 126, 153, 124, and 95, accounting for 28.12 % of the average annual number of universities in China. Furthermore, the number of university accidents in these provinces was 23, 23, 19, 29, 18, and 20 respectively, accounting for 53.23 % of university accidents in China.

The average annual GDP (100 billion RMB) of less economically developed regions, such as Yunnan Province, Tibet Autonomous Region, Gansu Province, Qinghai Province, Ningxia Hui Autonomous Region, and Xinjiang Uygur Autonomous Region, is 22.86, 1.72, 8.68, 2.90, 3.79 and 13.47, which accounts for 5.48% of China's average annual GDP. The average annual student population (10,000) is 85.26, 3.69, 52.74, 7.17, 13.5, and 42.43, accounting for 6.74% of China's average annual student population. The average annual number of universities is 80, 7, 49, 12, 19, and 53, accounting for 8.17% of the average annual number of universities in China. Of these, only 1 university accident was reported in Gansu Province during the entire period; the remaining provinces did not report university accidents, accounting for 0.40% of university accidents in China. The number of university accidents is characterized by a high incidence at high average annual GDP, average annual student population, and average annual number of universities, which may be due to the fact that the number of universities and the number of students tend to be higher in the developed regions than in the less developed regions, and therefore these three factors should be taken into account as influencing the incidence of accidents at universities.

### 3.3 Characteristics of the qualifications

Statistics on university accidents with different qualifications for 2017–2021 are shown in Table 1. The induced frequencies of fire mishaps, laboratory accidents, unintentional mishaps, suicide and homicide accidents for undergraduate and postgraduate students over the whole period were 70, 2, 29, 54; 11, 8, 7, 25. The undergraduate accident rates were 86 %, 20 %, 81 %, and 68 % and the postgraduate accident rates were 14 %, 80 %, 19 %, and 32 %, giving an overall undergraduate and postgraduate accident rate of 75.24 % and 24.76 % respectively. The rate of laboratory accidents is 4 times higher for postgraduates than for undergraduates, and the rate of fire mishaps, unintentional mishaps, suicide and homicide accidents is more than 2 times higher for undergraduates than for postgraduates. Overall, the number of accidents induced by undergraduate students is much higher than that of postgraduate students, and accidents show a significant differential distribution across qualifications, so qualifications should be considered as a factor in accident prevention.

## 4 Analysis of the influence strength values of university accidents

### 4.1 Analysis of time influence strength values

#### 4.1.1 Difference analysis of policies and semesters

**Fig. 6:**
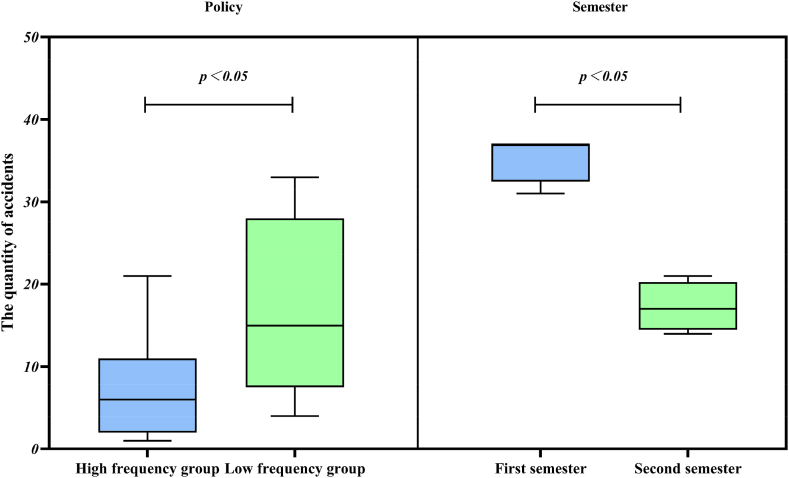
Chart of accident differences by policy and semester.

Based on the results of the descriptive statistical analysis, the number of policies was divided into high-frequency groups (number ≥77) and low-frequency groups (number <77), and March–June and September–December were divided into two groups: the second semester and the first semester. The high-frequency and low-frequency groups included 11 cases and 9 cases of different types of university accidents. The number of 4-month university accidents was included in both semester 2 and semester 1. Separate independent-sample T-tests were conducted for policy and semester, and the results are shown in Fig. 6.

The figure shows that the upper and lower quartiles and medians for the high and low-frequency groups are 11, 2, and 6; 28, 7.5, and 15, respectively. The upper and lower quartiles and medians for the first and second semesters were 37, 32.5, 37; 20.25, 14.5, and 17 respectively. Further, there was a statistically significant difference in the number of university accidents between policies and semesters (p < 0.05).

#### 4.1.2 Logistic regression models for policy and semester

**Fig. 7:**
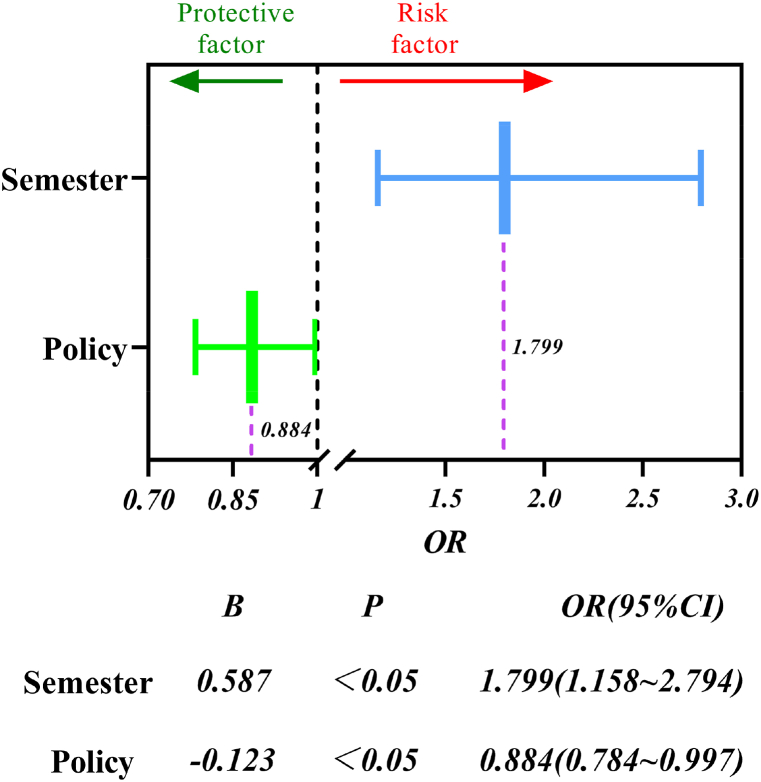
Semester and policy forest map.

Logistic regression analyses were performed on the policies and semesters of the different groups according to equations (1), (2), (3), (4), (5), (6), and the results are shown in Fig. 7.

The graph shows that different semesters significantly and positively affect the frequency of accidents in the university (B > 0) and OR = 1.799 > 1 i.e. the first semester has a value of 1.799 times the strength of the influence on the occurrence of accidents as compared to the second semester and is a risk factor. Policies in different frequency groups significantly and negatively affected the frequency of accidents at the University (B < 0), OR = 0.884 < 1 i.e. the strength of the influence of the high-frequency group on the occurrence of accidents was valued at only 88.4% of that of the low-frequency group and was a protective factor. The results of the regression analysis were statistically significant in both groups (p < 0.05).

#### 4.1.3 Diagnostic analysis of ROC curves for policies and semesters

**Fig. 8:**
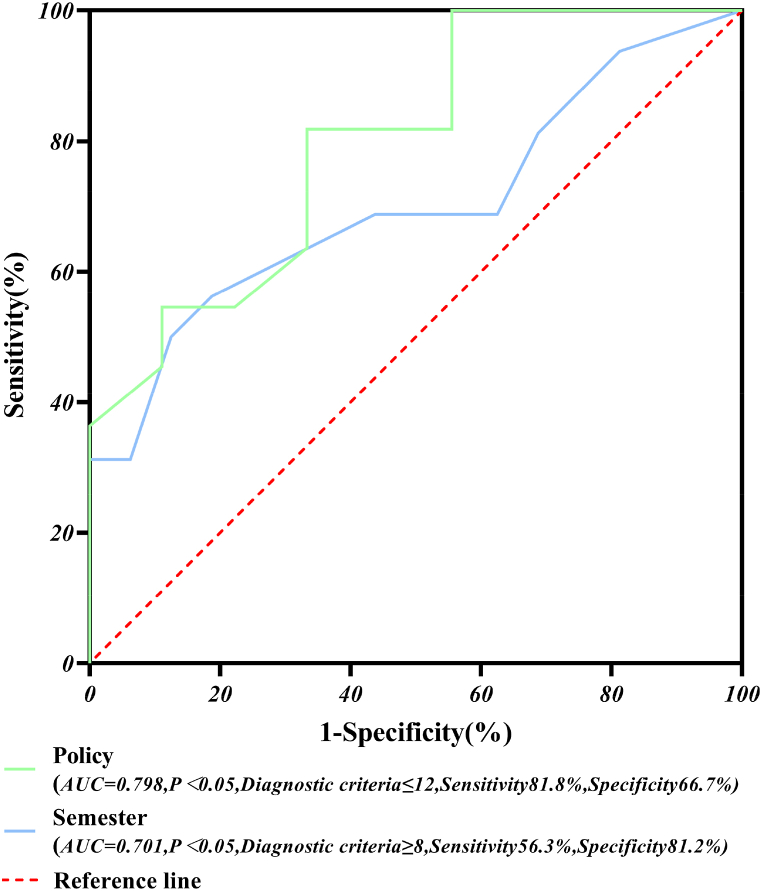
ROC curve diagnostic chart for policy and semester.

According to the results of the logistic regression model and equations (7), (8), (9), the policies and semesters were analyzed for the diagnostic analysis of the ROC curves, and the results are shown in Fig. 8.

The ROC curves were analyzed to show that the AUC and diagnostic criteria were 0.798, 0.701; ≤12, ≥8, for policy and semester, respectively. That is, the diagnostic accuracy for different numbers of policies and semesters was 79.8 % and 70.1 %, and a frequency of ≤12 for a certain type of incident (fire mishaps, laboratory accidents, unintentional mishaps, suicide and homicide accidents) was considered to be in the high-frequency group for that year's number of policies (or a frequency of ≥8 for a certain type of incident was considered to be in the first semester). The sensitivity and specificity of the policies and semesters were 81.8 % and 66.7 %; 56.3 % and 81.2 %, and the results of the diagnostic analysis of the ROC curves were statistically significant (P < 0.05).

### 4.2 Analysis of space influence strength values

#### 4.2.1 Difference analysis of average annual data by provinces

**Fig. 9:**
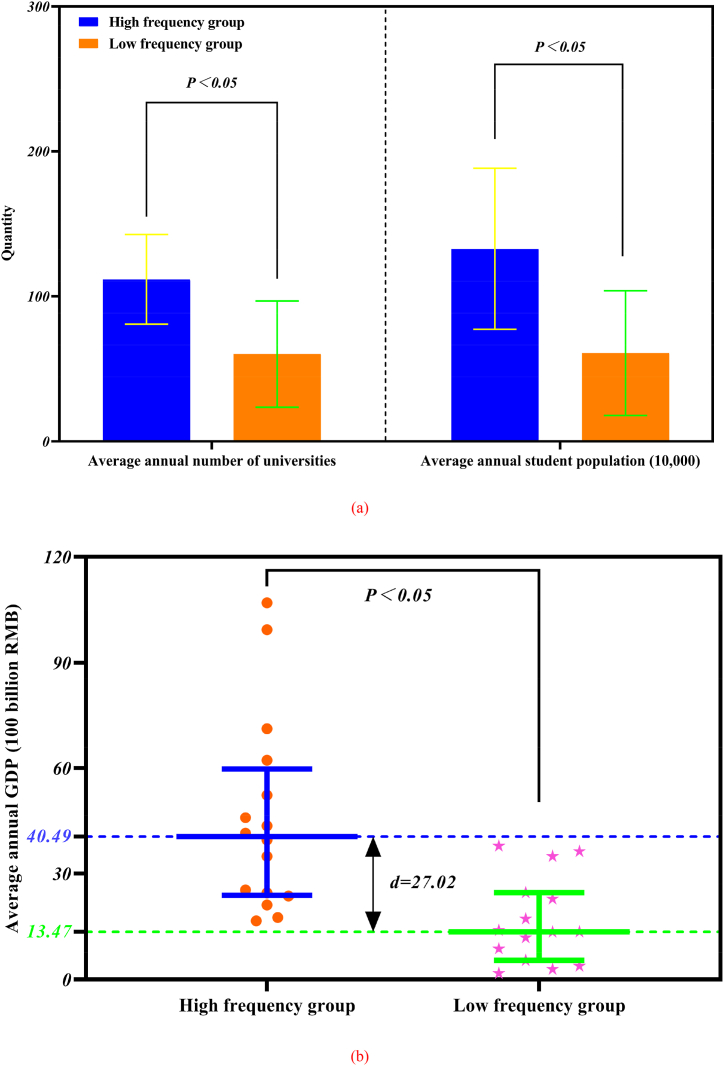
Differences in annual average data by province (a) show the average annual number of universities and average annual student population in each province, (b) shows the average annual GDP of each province.

Based on the results of descriptive statistical analyses, the number of accidents in each province was divided into a high-frequency group (≥6) and a low-frequency group (<6), of which 16 provinces were included in the high-frequency group and 15 in the low-frequency group. Independent-samples T-test for the average annual number of universities, average annual student population (10,000), and Mann-Whitney *U* test for average annual GDP (100 billion RMB), the results are shown in Fig. 9 (a, b).

The graph shows that the average annual number of universities and the average annual student population (10,000) in the high and low-frequency groups are 111.79, 60.20; 132.80, and 60.84. The upper and lower quartiles and medians of the high and low-frequency groups of average annual GDP (100 billion RMB) are 59.74, 23.86, 40.49; 24.60, 5.36, and 13.47 respectively. The difference in the number of university accidents was statistically significant (p < 0.05) for the average annual number of universities, the average annual student population (10,000), and the average annual GDP (100 billion RMB).

#### 4.2.2 Logistic regression models for average annual data by provinces

**Fig. 10:**
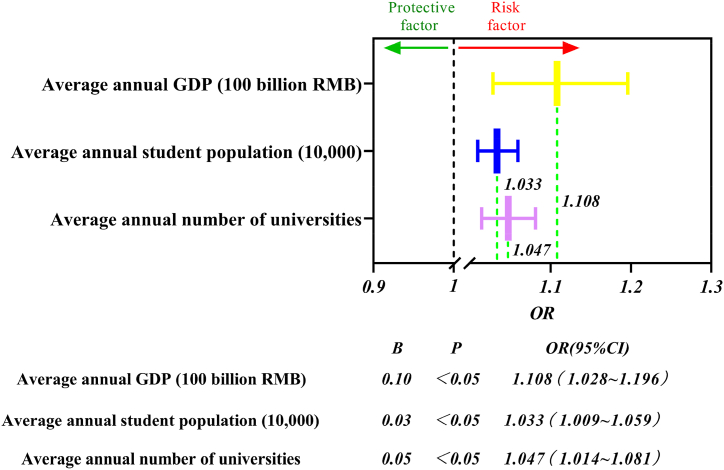
Average annual data forest map by province.

Logistic regression analysis was performed on the average annual data for provinces in the different groups according to equations (1), (2), (3), (4), (5), (6), and the results are shown in Fig. 10.

The figure shows that the average annual GDP (100 billion RMB), average annual student population (10,000), and the average annual number of universities significantly and positively influence the frequency of university accidents (B > 0), with OR values of 1.108, 1.033, and 1.047, respectively i.e., all three are risk factors. It means that a province with a high average annual GDP of 100 billion RMB is 1.108 times more likely to have an influence on the occurrence of accidents than a low 100 billion RMB; a province with a high average annual student population of 10,000 is 1.033 times more likely to have an influence on the occurrence of accidents than a low 10,000; and a province with a high average annual number of universities is 1.047 times more likely to have an influence on the occurrence of accidents than a low 1 university. The results of regression analyses of the three groups were statistically significant (P < 0.05).

#### 4.2.3 Diagnostic analysis of ROC curves for average annual data by provinces

**Fig. 11:**
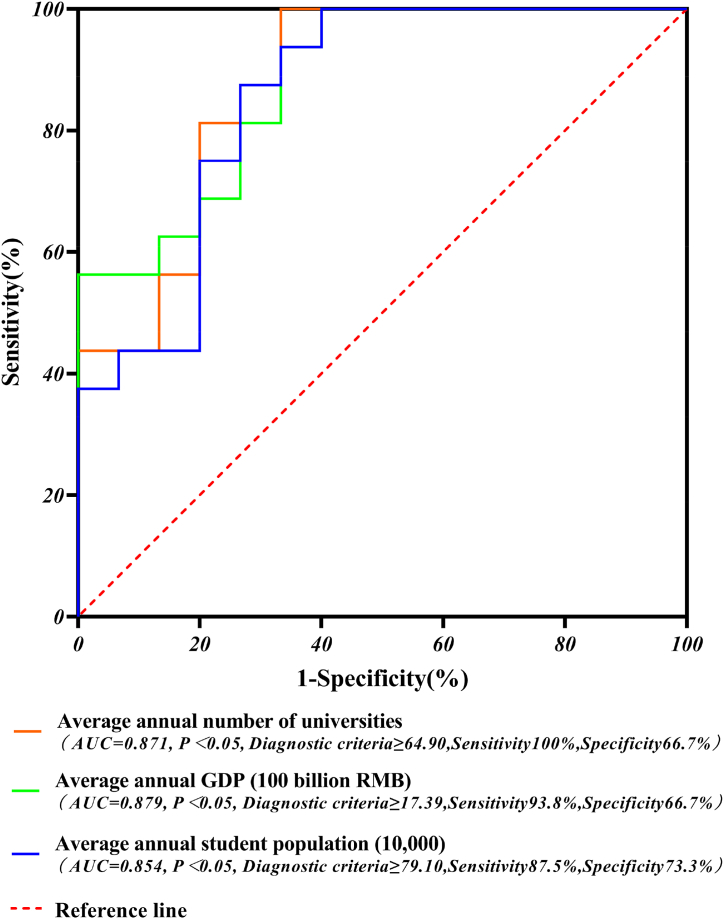
ROC curve diagnostic chart for average annual data by province.

Based on the results of the logistic regression model and equations (7), (8), (9), the average annual data of each province were analyzed by the diagnostic analysis of the ROC curves, and the results are shown in Fig. 11.

The ROC curves analysis showed that the AUC and diagnostic criteria for the average annual GDP (100 billion), the average annual student population (10,000), and the average annual number of universities were 0.879, 0.854, and 0.871; ≥17.39, ≥79.10, and ≥64.90, respectively; i.e. the diagnostic accuracy for all three was 87.9%, 85.4%, and 87.1%, respectively. When a province's average annual GDP (100 billion RMB) is ≥ 17.39 (average annual student population (10,000) ≥ 79.10 or the average annual number of universities (≥64.90)) it is considered to be prone to university accidents. Moreover, the sensitivity and specificity of the three groups were 93.8 %, 66.7 %; 87.5 %, 73.3 %; and 100 %, 66.7 %, and the results of the diagnostic analysis of the ROC curves were statistically significant (P < 0.05).

### 4.3 Analysis of qualifications influence strength values

#### 4.3.1 Differential analysis of qualifications

**Fig. 12:**
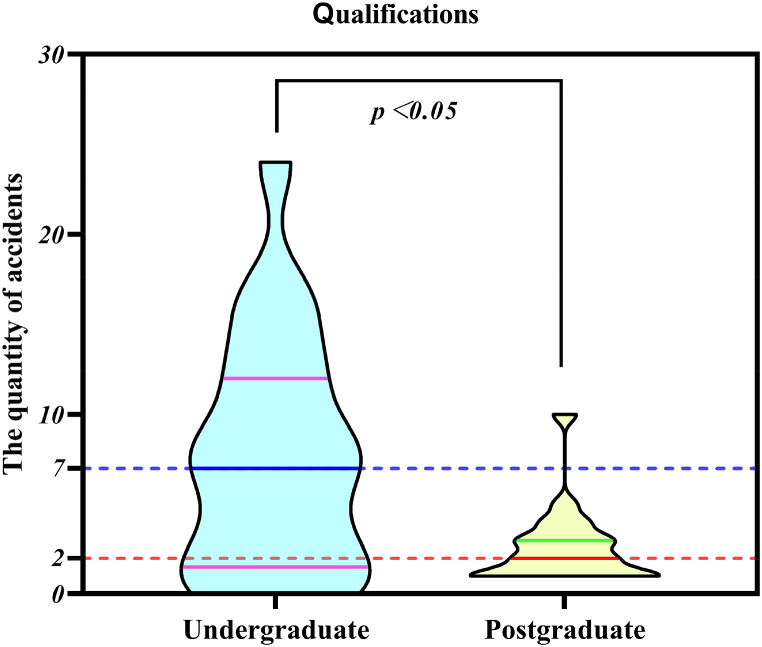
Chart of qualifications.

Based on the results of the descriptive statistical analysis, the causers were classified into undergraduate and postgraduate students according to their qualifications, where undergraduate and postgraduate students were included in 20 cases of different university accident types. The Mann-Whitney *U* test was performed on the qualifications and the results are shown in Fig. 12.

The figure shows that the upper and lower quartiles and medians of undergraduate and postgraduate qualifications are 12, 1.5, and 7; 3, 1, and 2, respectively, and that there is a statistically significant difference in the number of university accidents by qualification (p < 0.05).

#### 4.3.2 Logistic regression model of qualifications

**Fig. 13:**
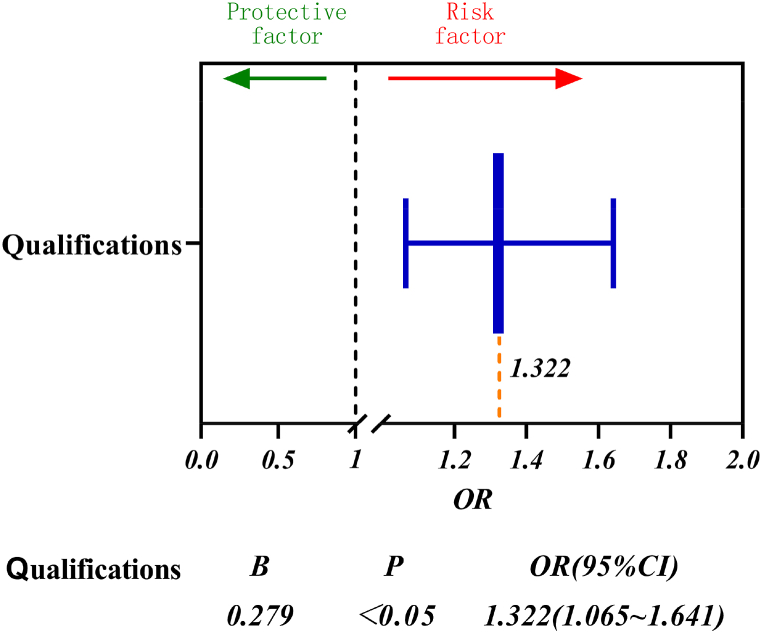
Qualifications forest map.

Logistic regression analyses were performed for different qualifications according to equations (1), (2), (3), (4), (5), (6) and the results are shown in Fig. 13.

The figure shows that different qualifications significantly and positively affect the frequency of accidents at the university (B > 0), OR = 1.322 > 1 i.e. the value of the strength of the influence of the undergraduate students on the occurrence of accidents is 1.322 times higher than that of the postgraduate students and is a risk factor. In addition, the results of regression analysis were statistically significant (p < 0.05).

#### 4.3.3 Diagnostic analysis of the ROC curves for qualifications

**Fig. 14:**
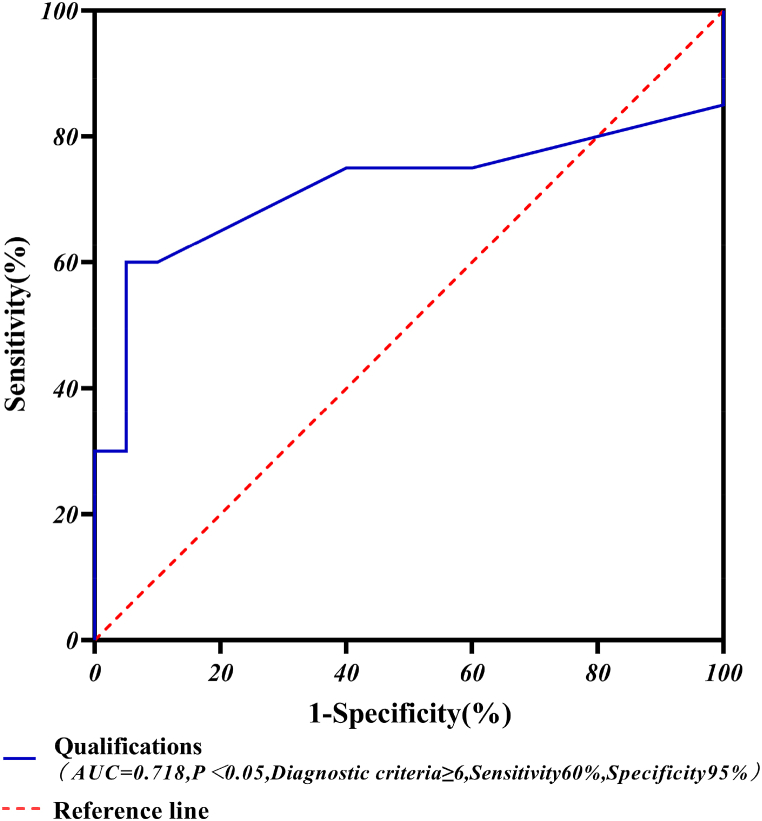
Diagnostic chart of ROC curve for qualifications.

According to the results of the logistic regression model and equations (7), (8), (9), the qualifications were analyzed for the diagnostic analysis of the ROC curve and the results are shown in Fig. 14.

The ROC curve analysis showed that the AUC and diagnostic criteria for qualifications were 0.718; ≥6, i.e. 71.8 % diagnostic accuracy across qualifications, respectively. The frequency of certain types of accidents (fire mishaps, laboratory accidents, unintentional mishaps, suicide and homicide accidents) ≥ 6 is considered to be the perpetrator of such accidents is an undergraduate student. Furthermore, the sensitivity and specificity of qualifications were 60 % and 95 %, and the results of the diagnostic analysis of ROC curves were statistically significant (P < 0.05).

## 5 Discussions

Given that university accidents in China show significant variability across time, space, and qualifications, as well as the identified influence strength values. Therefore, measures need to be taken to reduce its frequency and severity. This section provides appropriate recommendations to prevent and reduce these four types of accidents, namely, fire mishaps, laboratory accidents, unintentional mishaps, suicide and homicide accidents and discusses the laws and regulations governing safety on Chinese university campuses.

### 5.1 Fire mishaps

Fire mishaps accounted for 49.19 % of the number of accidents during the entire period, so the prevention and treatment of such accidents should be a priority to reduce casualties and property damage. According to the characteristics of the distribution of fire mishaps in time, space and qualifications should be strengthened in March–June and September–December each year for undergraduates in the relatively economically developed areas of fire prevention education. Throughout the period, student dormitories accounted for the vast majority of overall fire mishaps, so they should be made a priority site for fire prevention. Among all the factors that induce accidents, the human factor is the heaviest factor [37], so dormitory fire safety education should be carried out for students, such as: teaching the use of fire extinguishers, the determination of emergency escape routes, the use of high-power electrical equipment in the dormitory is strictly prohibited, smoking and so on. In addition, few automatic or manual fire extinguishing facilities are available to deal with accidents when they occur. Therefore, strengthening fire extinguishing facilities (e.g., smoke alarms, fire shutters, fire sprinklers, fire extinguishers, etc.) in student dormitories is also a key to solving fire accidents.

### 5.2 Laboratory accidents

Laboratory accidents are the least frequent accidents in the whole period with only 10 accidents, however, the number of casualties is only 1 person different from the number of casualties in fire mishaps which are the most frequent accidents, therefore, the university laboratory accidents should be given a sufficient level of attention. The distribution characteristics of laboratory accidents in time and space are not significant, but it is far more frequent in postgraduate qualifications than undergraduate qualifications, so postgraduate students should be the priority population for the prevention of laboratory accidents. In addition, laboratories often lack comprehensive supervisory and management mechanisms for individual behaviors, and the number of laboratory accidents caused by human factors is the highest, Mingqi Bai and Tomasz Olewski have both proposed to apply the principles of process safety management to university laboratories [11,12]. Therefore, reasonable safety management mechanisms should be developed for postgraduate students, such as the implementation of safety training, safety education, and risk assessment of unsafe behaviors. Reasonable safety management mechanisms enable students to familiarise themselves with the operating procedures of instruments and the various properties of experimental materials, as well as to identify various hazards in the laboratory and improve their emergency response capabilities for the purpose of preventing and reducing laboratory accidents.

### 5.3 Unintentional mishaps

Throughout the period, unintentional mishaps showed a high incidence in the three dimensions of September–December, economically developed regions, and undergraduate students. This may be due to the fact that two of the months from September to December are the hot season in China and that the number of universities and undergraduate students in the relatively developed regions of China is much higher than in the less developed regions. In addition, sudden deaths accounted for 63.16% of accidents in the entire period, with the number of sudden deaths being 22 while the rate of sudden deaths reached 90% so the prevention of sudden deaths should be a priority in the prevention of Unintentional mishaps. The AED can greatly improve patient survival, but only two patients were successfully saved throughout the survey period, suggesting that a very small percentage of universities have AED that can be quickly applied to patients. Therefore, in response to this phenomenon, every university in China should be equipped with AED as much as possible and have specialized people skilled in their use so that they can respond quickly to emergencies. In addition to the provision of AED, students should also be provided with campus safety education and publicity on how to deal with emergencies, so as to raise students' awareness of campus safety.

### 5.4 Suicide and homicide accidents

Suicide and homicide rates were high throughout the period, with higher rates in economically developed regions and among undergraduates, which may be due to similar causes as those of unintentional mishaps. The mortality rate of suicides and homicides accidents is nearly 100%, so in view of the seriousness of the consequences of such accidents, in addition to educating students about campus safety and publicizing ways of dealing with emergencies, it is also important to pay more attention to students' mental health on a daily basis and provide timely counseling or psychological intervention for students with mental health problems. Finally, the basic safety facilities of the university should be upgraded and full-time campus patrols should be carried out to further prevent and reduce the occurrence of accidents.

### 5.5 Lawmaking

Laws and regulations act as the only protective factor against university accidents throughout the period and are powerful in preventing and reducing all kinds of university accidents. In 2017 the General Office of the Ministry of Education of the People's Republic of China issued a circular on the issuance of the Guidelines on Fire Safety in Universities. In 2017, the Department of Science and Technology of the Ministry of Education of the People's Republic of China issued a circular on the safety inspection of university research laboratories 2017. In 2019 the Ministry of Education of the People's Republic of China issued the Opinions on Strengthening the Safety of University Laboratories. In 2021, the General Office of the Ministry of Education of the People's Republic of China issued a circular on the implementation of special actions to strengthen the safety of university laboratories. In general, the Chinese government has increased the importance of university accidents, but the laws and regulations on university accidents in China are not yet systematic and are only at the level of opinions or circulars. Compared with other safety fields, it is still in the beginning stage and has much room for progress, such as the laws and regulations related to hazardous chemicals have changed from rough type to refined construction [38]. The building of laws and regulations helps to develop a safety culture at the university, which serves to continually raise safety standards by reinforcing individual safety concepts, attitudes, emotions, behaviors, morals, and ethics, as well as through leadership, education, communication, rewards and sanctions [39]. Therefore, the Chinese Government should establish targeted laws and regulations in response to various university accidents, which will help to form a university safety culture and better enable students to actively participate in relevant safety matters, assume responsibility for safety, and strengthen their attitudes and behaviors with regard to safety on university campuses, thus further forming a good safety awareness and behavioral habits.

## 6 Conclusions

Due to the frequent occurrence of university accidents in China, there are numerous student casualties. Therefore, it has become a priority for the government and school administrators to explore the relationship patterns of the factors influencing accidents, and to prevent and reduce the occurrence of accidents. This paper presents a mathematical analysis model based on 248 university accidents that occurred in China from 2017 to 2021. The first stage of the model is the difference analysis, which analyses the data using independent-sample T-tests and the Mann-Whitney *U* test to assess whether the differences in the number of university accidents with different influencing factors are statistically significant. The second stage of the model is to perform Logistic regression analyses on the different influencing factors that are statistically significant to determine the influence strength values of the influencing factors on the accidents. The third stage of the model is to diagnose the results of the logistic regression analysis with the ROC curves to determine the accuracy of the logistic regression analysis. The numerical results derived from the above three steps show that the mathematical analysis model accurately determines the relationship pattern of the factors influencing the accidents. The results are as follows:

● University accidents are significantly affected by different times, the high-frequency policy (protective factors) has only 88.4 % of the strength value of the low-frequency influence on the occurrence of accidents, and the first semester (risk factors) has 1.799 times the strength value of the second-semester influence on the occurrence of accidents, and the model diagnostic accuracy is 79.8 % and 70.1 %, respectively.
● Different spaces significantly affect university accidents and are all risk factors, with provinces with a high average annual GDP of 100 billion RMB having 1.108 times the strength of influence on the occurrence of accidents than those with a low average GDP of 100 billion RMB. Provinces with 10,000 higher average annual student populations have 1.033 times the strength of influence on the occurrence of accidents than provinces with 10,000 lower average annual student populations. The value of the strength of the influence on the occurrence of accidents in provinces with 1 higher average annual number of universities is 1.047 times higher than in provinces with 1 lower average number of universities. The model diagnostic accuracies were 87.9 %, 85.4 %, and 87.1 %, respectively.
● Different qualifications had a significant effect on university accidents, with undergraduate students having a value of 1.322 times the strength of the influence on the occurrence of accidents as compared to postgraduate students and as a risk factor, and the model had a diagnostic accuracy of 71.8%.

Therefore, the future prevention and reduction of accidents at the University, based on the relationship patterns of the factors influencing accidents, should be focused on the following three directions:

● Establishment of laws and regulations. Developing more refined rules and regulations for university accidents, for example, promulgating some standards and regulations for laboratory accidents in terms of laboratory design, laboratory acceptance, laboratory risk assessment, and laboratory production accountability.
● Optimizing management techniques. Students are given increased awareness of safety on university campuses, regular surveys on student mental health, and basic safety facilities are equipped or upgraded.
● Government and university administrators should increase their focus on university accidents. The government and university administrators should organize regular educational lectures on campus safety by specialists, draw up contingency plans for accidents and conduct regular drills, etc.

However, this study has some limitations that need to be addressed in future studies. Firstly, the raw data for the study was only for accidents during the 5 years from 2017 to 2021, and future studies should encompass more accidents by increasing the time period of accidents, e.g. 10 or 20 years. Second, there is no specialized agency in China responsible for counting and publishing university accidents, and only accidents reported by authoritative media and databases were used in this study. Therefore, as many media websites as possible, including non-authoritative media websites and databases, should be investigated in future studies to increase the sample data. Thirdly, this study only analyzed the influence of time, space, and qualification on university accidents. Therefore, future research should be conducted on more specific influencing factors, such as whether or not students have received university safety education and whether or not the university has an emergency plan for accidents.

In conclusion, this study provides valuable insights into the prevention and reduction of accidents at the University. Considers the influence of three dimensions of time, space, and qualification on university accidents and is able to accurately determine the relationship pattern of factors influencing accidents. Future research should address the limitations presented as a means of further grasping the development pattern of accidents at the University and making more specific and effective recommendations for accident prevention and reduction.

## Author contribution statement

Guixiang Wu: Conceived and designed the experiments.

Yanfei Yang: Performed the experiments; Analyzed and interpreted the data; Wrote the paper.

Chenglin Xu: Contributed reagents, materials, analysis tools or data.

## Data availability statement

Data will be made available on request.

## Funding statement

This paper was supported by the Yunnan Provincial Key R&D Program Social Development Special, （NO.202003AC100002）

## Declaration of competing interest

The authors declare that they have no known competing financial interests or personal relationships that could have appeared to influence the work reported in this paper.
