# Supplementary material for: Characteristics and statistical analysis of university accidents in China from 2017 to 2021
Source: Heliyon. 2023 Oct 11;9(10):e20616. doi: 10.1016/j.heliyon.2023.e20616 (PMC10590799; doi:10.1016/j.heliyon.2023.e20616)
Supplement: Multimedia component 1 [file mmc1.docx]

**Supporting Information**

**Characteristics and Statistical Analysis of University Accidents in China from 2017 to 2021**

Guixiang Wu*, Yanfei Yang, Chenglin Xu

School of public safety and emergency management， Kunming University of Science and Technology， Kunming Yunnan 650093, PR China

Email: [20212239045@stu.kust.edu.cn](mailto:20212239045@stu.kust.edu.cn)

# 1. Methods and Results of the 2017–2021 China University Accidents Survey

A separate government organization to compile statistics and publish university accidents has not yet been established in China. The data used in this essay is primarily taken from many authoritative Chinese websites and articles that have been published in top academic journals worldwide.

- **Using a manual search engine, look up accident statistics in literature and on the web**

We started by looking for accidents involving university safety on the website of the People's Republic of China's Ministry of Emergency Management, People's Daily, Xinhua, Sohu News, and Baidu News. We then included accidents that resulted in fatalities or property damage in the data. Accident statistics are made easier by specific subject phrases, hence this article uses the following search subject terms: China University Fire mishaps 2017, China University Laboratory accidents 2017, China University Unintentional mishaps 2017, China University Suicide and homicide accidents 2017; China University Fire mishaps 2018, China University Laboratory accidents 2018, China University Unintentional mishaps 2018, China University Suicide and homicide accidents 2018; China University Fire mishaps 2019, China University Laboratory accidents 2019, China University Unintentional mishaps 2019, China University Suicide and homicide accidents 2019; China University Fire mishaps 2020, China University Laboratory accidents 2020, China University Unintentional mishaps 2020, China University Suicide and homicide accidents 2020; China University Fire mishaps 2021, China University Laboratory accidents 2021, China University Unintentional mishaps 2021, China University Suicide and homicide accidents 2021.

Then, databases such as Web of Science, ElsevierSD, SpringerLink, CNKI, and others were searched for literature relating to university accidents in China. The search subject terms were set: "accidents in Chinese universities," "fire mishaps in Chinese universities," "laboratory accidents in Chinese universities," "Unintentional mishaps in Chinese universities," and "Suicide and homicide accidents in Chinese universities," all in English, covering any genre of literature, from January 1, 2017, to December 31, 2021.

- **Data Screening**

This paper manually checks the counted Chinese university accidents to ensure the accuracy and dependability of the data. To remove fabricated accidents involving Chinese universities, the screening process mostly involved verifying the incident's date and location. Finally, data on 248 accidents at Chinese universities between 2017 and 2021 was obtained. Detailed statistical results are shown in Table s1.

**Table s1.** University accidents in China, 2017-2021

| Accident  Number | Date | Location | Accident  Type | Description | Consequences | Source |
| --- | --- | --- | --- | --- | --- | --- |
| 1 | 10/9/2017 | South China Agricultural University | Fire mishaps | Dormitory fire | 0 Fatalities/  0 Injuries/ Facilities  Damage | NetEase News  <https://www.163.com/dy/article/GLVTV7OO0536NCEV.html> |
| 2 | 8/14/2017 | Guangxi University | Fire mishaps | Dormitory fire | 0 Fatalities/  0 Injuries/ Facilities  Damage | Sohu News  <https://www.sohu.com/a/494180849_121124565> |
| 3 | 5/17/2017 | Tsinghua University | Fire mishaps | Dormitory fire | 0 Fatalities/  0 Injuries/ Facilities  Damage | Sohu News  <https://www.sohu.com/a/141170754_765855> |
| 4 | 9/4/2017 | Swan College, Central South University of Forestry and Technology | Fire mishaps | Dormitory fire | 0 Fatalities/  0 Injuries/ Facilities  Damage | Sohu News  <https://www.sohu.com/a/190706345_390667> |
| 5 | 10/31/2017 | Hunan City University | Fire mishaps | Dormitory fire | 0 Fatalities/  0 Injuries/ Facilities  Damage | Sohu News  <https://m.sohu.com/a/201526566_369029/> |
| 6 | 5/15/2017 | Tianjin University of Traditional Chinese Medicine | Fire mishaps | Dormitory fire | 0 Fatalities/  0 Injuries/ Facilities  Damage | National Emergency Broadcasting Network  <http://www.cneb.gov.cn/2017/05/16/ARTI1494900508326480.shtml> |
| 7 | 3/15/2017 | Shangrao Normal University | Fire mishaps | Dormitory fire | 0 Fatalities/  0 Injuries/ Facilities  Damage | National Emergency Broadcasting Network  <http://www.cneb.gov.cn/2017/03/14/ARTI1489452312140529.shtml> |
| 8 | 4/4/2017 | Beijing University of Posts and Telecommunications | Fire mishaps | Network Data Center Fires | 0 Fatalities/  0 Injuries/ Facilities  Damage | China News Network  <http://jiangsu.china.com.cn/html/edu/info/10069065_1.html> |
| 9 | 12/8/2017 | Chongqing Jiaotong University | Fire mishaps | Academic building fire | 0 Fatalities/  0 Injuries/ Facilities  Damage | Sina News  <http://cq.sina.com.cn/news/s/2017-12-08/detail-ifypnsin9839892.shtml> |
| 10 | 4/16/2017 | Chongqing Technology and Business University | Fire mishaps | Academic building fire | 0 Fatalities/  0 Injuries/ Facilities  Damage | Baidu News  <https://tieba.baidu.com/p/5074837655> |
| 11 | 5/15/2017 | Sichuan Conservatory of Music | Fire mishaps | Academic building fire | 0 Fatalities/  0 Injuries/ Facilities  Damage | Sohu News  <https://www.sohu.com/a/140766217_796783> |
| 12 | 3/18/2017 | North University of China | Fire mishaps | Laboratory building fire | 0 Fatalities/  0 Injuries/ Facilities  Damage | Sohu News  <https://www.sohu.com/a/129351251_260616> |
| 13 | 7/10/2017 | Xi’an Polytechnic University | Fire mishaps | Dormitory fire | 0 Fatalities/  0 Injuries/ Facilities  Damage | Sohu News  <https://www.sohu.com/a/156455969_384315> |
| 14 | 3/29/2017 | Xi’an University of Technology | Fire mishaps | Dormitory fire | 0 Fatalities/  0 Injuries/ Facilities  Damage | Sohu News  <https://www.sohu.com/a/131079725_384315> |
| 15 | 3/18/2017 | Beijing Institute of Graphic Communication | Fire mishaps | Pile fires | 0 Fatalities/  0 Injuries/ Facilities  Damage | Sina News  <https://zx.sina.cn/n/2017-03-18/zx-ifycnpiu9018865.d.html?vt=4&wm=3049_0018> |
| 16 | 4/24/2017 | Chang’an University | Fire mishaps | Dormitory fire | 0 Fatalities/  0 Injuries/ Facilities  Damage | Yanshan University Safety Work Office  <http://bwc.ysu.edu.cn/info/2022/3178.htm> |
| 17 | 5/23/2017 | University of Science and Technology Beijing | Fire mishaps | Academic building fire | 0 Fatalities/  0 Injuries/ Facilities  Damage | Yanshan University Safety Work Office  <http://bwc.ysu.edu.cn/info/2022/3178.htm> |
| 18 | 6/18/2017 | Tsinghua University | Fire mishaps | Laboratory building fire | 0 Fatalities/  0 Injuries/ Facilities  Damage | Yanshan University Safety Work Office  <http://bwc.ysu.edu.cn/info/2022/3178.htm> |
| 19 | 6/12/2017 | Unannounced | Fire mishaps | Unannounced | 0 Fatalities/  0 Injuries/ Facilities  Damage | CCTV  <http://tv.cctv.com/2017/06/12/VIDEnFdQcjOU6SwINysePCM8170612.shtml> |
| 20 | 12/8/2017 | Zhejiang Ocean University | Fire mishaps | Pile fires | 0 Fatalities/  0 Injuries/ Facilities  Damage | College of Marine Science and Technology, Zhejiang Ocean University  <http://msc.zjou.edu.cn/info/1152/2438.htm> |
| 21 | 3/27/2017 | Fudan University | Laboratory accidents | Laboratory explosion | 0 Fatalities/  1 Injury/ Facilities  Damage | Nanjing Information Engineering University Laboratory Safety Education and Management Network  <http://examsafety.nuist.edu.cn/redir.php?catalog_id=137&object_id=18279> |
| 22 | 3/18/2017 | North University of China | Laboratory accidents | Laboratory fires | 0 Fatalities/  0 Injuries/ Facilities  Damage | Sohu News  <https://www.sohu.com/a/129351251_260616> |
| 23 | 1/10/2017 | Tsinghua University | Unintentional mishaps | Overboard | 1 Fatality/  1 Injury | Beijing Institute of Petrochemical Technology  <https://www.bipt.edu.cn/publish/aqscyjy/jyxx/102544.htm> |
| 24 | 11/6/2017 | Guangxi Water Conservancy and Electricity Vocational and Technical College | Unintentional mishaps | Falling from a height | 1 Fatality | Guiyang Evening News  <https://baijiahao.baidu.com/s?id=1585213526524032783&wfr=spider&for=pc> |
| 25 | 12/26/2017 | Guangdong University of Technology | Unintentional mishaps | Falling from a height | 1 Fatality | NetEase News  <https://www.163.com/dy/article/D6K954LU05129QAF.html> |
| 26 | 2/17/2017 | Guangxi University | Unintentional mishaps | sudden death | 1 Fatality | China News Network  <http://jiangsu.china.com.cn/html/2017/gxnews_0221/9432985.html> |
| 27 | 12/9/2017 | Shanxi Agricultural University | Unintentional mishaps | sudden death | 1 Fatality | NetEase News  <https://www.163.com/dy/article/D632D6300512808N.html> |
| 28 | 11/19/2017 | Wuhan University | Unintentional mishaps | sudden death | 1 Fatality | Sohu News  <https://www.sohu.com/a/349487075_689780> |
| 29 | 3/22/2017 | Southern Medical University | Suicide and homicide accidents | Homicide accidents | 2 Fatalities | Yanshan University Safety Work Office  <http://bwc.ysu.edu.cn/info/2022/3178.htm> |
| 30 | 7/6/2017 | Tianjin University of Technology and Education | Suicide and homicide accidents | Suicide accidents | 1 Fatality | NetEase News  <https://www.163.com/dy/article/DG75KFN50516A1OC.html> |
| 31 | 2/19/2017 | Sichuan Normal University | Suicide and homicide accidents | Suicide accidents | 1 Fatality | NetEase News  <https://m.163.com/dy/article/DF7AMK4H0525SNT7.html> |
| 32 | 3/4/2017 | Weinan Vocational and Technical College | Suicide and homicide accidents | Suicide accidents | 1 Fatality | China Youth Network  <http://edu.youth.cn/jyzx/jyxw/201703/t20170310_9260640.htm> |
| 33 | 1/11/2017 | Shandong University | Suicide and homicide accidents | Suicide accidents | 1 Fatality | CCTV  <http://news.cctv.com/2017/04/23/ARTIjAE2L0rYCJuFIEG5N07V170423.shtml> |
| 34 | 4/11/2017 | Xiamen Huaxia University | Suicide and homicide accidents | Suicide accidents | 1 Fatality | China Youth Network  <https://baijiahao.baidu.com/s?id=1564644309994773&wfr=spider&for=pc> |
| 35 | 2/27/2017 | Guangxi University | Suicide and homicide accidents | Suicide accidents | 1 Fatality | Science Network  <https://news.sciencenet.cn/htmlnews/2017/3/369160.shtm> |
| 36 | 12/25/2017 | Xi’an Jiaotong University | Suicide and homicide accidents | Suicide accidents | 1 Fatality | China Youth Network  <http://edu.youth.cn/jyzx/jyxw/201801/t20180110_11265752.htm> |
| 37 | 3/7/2017 | Nanning College for Vocational Technology | Suicide and homicide accidents | Suicide accidents | 1 Fatality | Sohu Network  <https://www.sohu.com/a/128559711_394145> |
| 38 | 5/8/2017 | Dongguan City Electronic Technology School | Suicide and homicide accidents | Suicide accidents | 1 Fatality | Anhui Net  <http://www.ahwang.cn/p/1632336.html> |
| 39 | 9/1/2017 | Shaanxi Aviation Vocational and Technical College | Suicide and homicide accidents | Suicide accidents | 1 Fatality | Sohu Network  <https://www.sohu.com/a/190678119_639898> |
| 40 | 6/29/2017 | Chongqing College of Mobile Communication | Suicide and homicide accidents | Suicide accidents | 1 Fatality | Sohu Network  <https://www.sohu.com/a/154702068_260616> |
| 41 | 4/18/2017 | Unannounced | Suicide and homicide accidents | Suicide accidents | 1 Fatality | Sohu Network  <https://www.sohu.com/a/134214240_735845> |
| 42 | 8/15/2017 | Unannounced | Suicide and homicide accidents | Suicide accidents | 1 Fatality | NetEase News  <https://www.163.com/dy/article/ECSG9CTA05218435.html> |
| 43 | 6/10/2017 | Northwestern Polytechnical University | Suicide and homicide accidents | Suicide accidents | 1 Fatality | Guangming Network  <https://m.gmw.cn/baijia/2018-01/13/27340190.html#verision=b92173f0> |
| 44 | 1/3/2018 | Beijing Normal University | Fire mishaps | Garden fire | 0 Fatalities/  0 Injuries/ Facilities  Damage | Weibo News  <https://weibo.com/2177386743/FCMciuJIW?type=comment> |
| 45 | 2/3/2018 | Dongguan Institute of Technology | Fire mishaps | Dormitory fire | 0 Fatalities/  0 Injuries/ Facilities  Damage | Tencent News  <https://gd.qq.com/a/20180205/007800.htm> |
| 46 | 10/25/2018 | Xi’an Jiaotong University | Fire mishaps | Dormitory fire | 0 Fatalities/  0 Injuries/ Facilities  Damage | North Youth Network  <https://t.ynet.cn/baijia/23133795.html> |
| 47 | 12/11/2018 | Unannounced | Fire mishaps | Canteen fire | 0 Fatalities/  0 Injuries/ Facilities  Damage | Sohu News  <https://learning.sohu.com/a/583191143_121106869> |
| 48 | 12/9/2018 | Unannounced | Fire mishaps | Canteen fire | 0 Fatalities/  0 Injuries/ Facilities  Damage | Inner Mongolia Morning Post  <https://baijiahao.baidu.com/s?id=1619530504571384869&wfr=spider&for=pc> |
| 49 | 4/13/2018 | Unannounced | Fire mishaps | Dormitory fire | 0 Fatalities/  0 Injuries/ Facilities  Damage | Baidu News  <https://mp.weixin.qq.com/s?__biz=MjM5OTA1OTkyNA==&mid=2654496777&idx=1&sn=720832a2b8851088a71f0429955cf911&chksm=bd0d80b28a7a09a4464ffbc232d876dc8b5cbd710a9f5395774f570411e798c7e84db0d4364e&scene=27> |
| 50 | 12/26/2018 | Beijing Jiaotong University | Fire mishaps | Laboratory building fire | 0 Fatalities/  0 Injuries/ Facilities  Damage | Xinhua News Agency  <https://baijiahao.baidu.com/s?id=1620882975054231665&wfr=spider&for=pc> |
| 51 | 8/21/2018 | Renmin University of China | Fire mishaps | Dormitory fire | 0 Fatalities/  0 Injuries/ Facilities  Damage | Sohu News  <https://www.sohu.com/a/249463363_476402> |
| 52 | 12/16/2018 | Beihang University | Fire mishaps | Canteen fire | 0 Fatalities/  0 Injuries/ Facilities  Damage | Sohu News  <https://www.sohu.com/a/283009669_120042918> |
| 53 | 10/8/2018 | Zhongyuan University of Technology | Fire mishaps | Library Fire | 0 Fatalities/  0 Injuries/ Facilities  Damage | Sohu News  <https://www.sohu.com/a/259222947_643923> |
| 54 | 10/11/2018 | Chengdu University of Technology | Fire mishaps | Supermarket fire | 0 Fatalities/  0 Injuries/ Facilities  Damage | Baidu News  <https://tieba.baidu.com/p/2008914930> |
| 55 | 9/4/2018 | Unannounced | Fire mishaps | Dormitory fire | 0 Fatalities/  0 Injuries/ Facilities  Damage | Sohu News  <https://www.sohu.com/a/202977828_400941> |
| 56 | 11/12/2018 | Jishou University | Fire mishaps | Dormitory fire | 0 Fatalities/  0 Injuries/ Facilities  Damage | Weibo News  <https://weibo.com/6073300935/H400vnFTt> |
| 57 | 12/27/2018 | Chongqing Institute of Engineering | Fire mishaps | Dormitory fire | 0 Fatalities/  0 Injuries/ Facilities  Damage | Sina News  <http://k.sina.com.cn/article_1686546714_6486a91a02000k7g2.html> |
| 58 | 5/11/2018 | Chongqing University of Posts and Telecommunications | Fire mishaps | Dormitory fire | 0 Fatalities/  0 Injuries/ Facilities  Damage | Tencent News  <https://cq.qq.com/a/20181230/001329.htm> |
| 59 | 12/30/2018 | Yulin University | Fire mishaps | Dormitory fire | 0 Fatalities/  0 Injuries/ Facilities  Damage | Sohu News  <https://www.sohu.com/a/344443474_99903639> |
| 60 | 6/10/2018 | Inner Mongolia Construction Vocational and Technical College | Fire mishaps | Canteen fire | 0 Fatalities/  0 Injuries/ Facilities  Damage | Sohu News  <https://www.sohu.com/a/281959767_120028877> |
| 61 | 12/9/2018 | Inner Mongolia University of Finance and Economics | Fire mishaps | Canteen fire | 0 Fatalities/  0 Injuries/ Facilities  Damage | Inner Mongolia Morning Post  <https://baijiahao.baidu.com/s?id=1619530504571384869&wfr=spider&for=pc> |
| 62-66 | 10/25/2018-12/4/2018 | Xi’an Jiaotong University | Fire mishaps | Dormitory fire | 0 Fatalities/  12 Injuries/ Facilities  Damage | Knowing the network  <https://www.zhihu.com/question/302004121> |
| 67 | 10/2/2018 | Xi’an University of Technology | Fire mishaps | Dormitory fire | 0 Fatalities/  0 Injuries/ Facilities  Damage | Tencent News  <https://v.qq.com/x/page/t08061063mb.html> |
| 68 | 12/7/2018 | Guizhou University of Engineering Science | Fire mishaps | Dormitory fire | 0 Fatalities/  0 Injuries/ Facilities  Damage | Weibo News  <https://weibo.com/2594225611/H68zwFAXr> |
| 69 | 11/5/2018 | Guizhou Minzu University | Fire mishaps | Building fires | 0 Fatalities/  0 Injuries/ Facilities  Damage | Global Network  <https://baijiahao.baidu.com/s?id=1616273200747359229&wfr=spider&for=pc> |
| 70 | 4/9/2018 | Guangxi Technological College of Machinery and Electricity | Fire mishaps | Dormitory fire | 0 Fatalities/  0 Injuries/ Facilities  Damage | Sohu News  <https://www.sohu.com/a/227792416_548964> |
| 71 | 12/24/2018 | Fujian Normal University | Fire mishaps | Dormitory fire | 0 Fatalities/  0 Injuries/ Facilities  Damage | China Youth Network  <https://baijiahao.baidu.com/s?id=1620727229664181180&wfr=spider&for=pc> |
| 72 | 12/8/2018 | Unannounced | Fire mishaps | Dormitory fire | 0 Fatalities/  2 Injuries/ Facilities  Damage | Weibo News  <https://weibo.com/1647210043/H6hPi7nie> |
| 73 | 12/26/2018 | Beijing Jiaotong University | Laboratory accidents | Laboratory explosion | 3 Fatalities/  Facilities  Damage | Current status, challenges, and future directions of university laboratory  safety in China  <https://doi.org/10.1016/j.jlp.2021.104671> |
| 74 | 11/11/2018 | Hanlin College of Nanjing University of Chinese Medicine | Laboratory accidents | Laboratory deflagration | 2 Fatalities/  9 Injuries/ Facilities  Damage | People's Daily News  <https://baijiahao.baidu.com/s?id=1616820922973332488&wfr=spider&for=pc> |
| 75 | 12/29/2018 | Guangdong University of Technology | Unintentional mishaps | Accidental fall and death | 1 Fatality | NetEase News  <https://m.163.com/all/article/E4820MF7000187VE.html> |
| 76 | 3/19/2018 | Guangxi University | Unintentional mishaps | sudden death | 1 Fatality | Guangxi News Network  <http://www.gxnews.com.cn/staticpages/20170220/newgx58aa1d69-15960024.shtml?pcview=1> |
| 77 | 4/26/2018 | Tianjin University | Unintentional mishaps | sudden death | 1 Fatality | NetEase News  <https://m.163.com/dy/article/DGGEC6H50513EF9U.html> |
| 78 | 5/5/2018 | Nanjing University Of Finance & Economics | Unintentional mishaps | sudden death | 1 Fatality | Baidu News  <https://baijiahao.baidu.com/s?id=1599770601654950197&wfr=spider&for=pc> |
| 79 | 11/11/2018 | Guangzhou University of Chinese Medicine | Unintentional mishaps | sudden death | 1 Fatality | Global Network  <https://baijiahao.baidu.com/s?id=1617541570293577121&wfr=spider&for=pc> |
| 80 | 1/2/2018 | Zhejiang Institute of Mechanical & Electrical Engineering | Unintentional mishaps | sudden death | 1 Fatality | Minnan Network  <http://www.mnw.cn/edu/xiaoyuan/1915352.html> |
| 81 | 6/28/2018 | Sichuan International Studies University | Unintentional mishaps | sudden death | 1 Fatality | Baidu News  <https://baijiahao.baidu.com/s?id=1605119001076964802&wfr=spider&for=pc> |
| 82 | 10/13/2018 | South China University of Technology | Suicide and homicide accidents | Suicide accidents | 1 Fatality | NetEase News  <https://www.163.com/dy/article/E3SA7TFN0519BMI1.html> |
| 83 | 3/26/2018 | Wuhan University of Technology | Suicide and homicide accidents | Suicide accidents | 1 Fatality | People's Daily News  <https://baijiahao.baidu.com/s?id=1629021681415877510&wfr=spider&for=pc> |
| 84 | 6/5/2018 | Wuhan University | Suicide and homicide accidents | Suicide accidents | 1 Fatality | Baidu News  <https://baijiahao.baidu.com/s?id=1603130126412549599&wfr=spider&for=pc> |
| 85 | 11/4/2018 | Hunan University of Information Technology | Suicide and homicide accidents | Suicide accidents | 1 Fatality | CCTV  <https://baijiahao.baidu.com/s?id=1617258752768383587&wfr=spider&for=pc> |
| 86 | 6/12/2018 | Guangxi Normal University | Suicide and homicide accidents | Suicide accidents | 1 Fatality | Sohu News  <https://www.sohu.com/a/235403993_652954> |
| 87 | 3/30/2018 | Hunan University | Suicide and homicide accidents | Suicide accidents | 1 Fatality | Xinjing News  <https://baijiahao.baidu.com/s?id=1596459876321737955&wfr=spider&for=pc> |
| 88 | 4/13/2018 | Ocean University of China | Suicide and homicide accidents | Suicide accidents | 1 Fatality | China Youth Network  <https://baijiahao.baidu.com/s?id=1598048118646363281&wfr=spider&for=pc> |
| 89 | 1/30/2018 | Hebei University of Architecture | Suicide and homicide accidents | Suicide accidents | 1 Fatality | Baidu News  <https://baijiahao.baidu.com/s?id=1593736074294795734&wfr=spider&for=pc> |
| 90 | 2/3/2018 | Wuhan University of Technology | Suicide and homicide accidents | Suicide accidents | 1 Fatality | Baidu News  <https://baijiahao.baidu.com/s?id=1591365292548964784&wfr=spider&for=pc> |
| 91 | 10/10/2018 | Zhejiang University | Suicide and homicide accidents | Suicide accidents | 1 Fatality | Global Network  <https://baijiahao.baidu.com/s?id=1614301689477433202&wfr=spider&for=pc> |
| 92 | 7/2/2018 | Sichuan International Studies University | Suicide and homicide accidents | Homicide accidents | 2 Fatalities | Weibo News  <https://weibo.com/5659237191/GoGsBFzcU> |
| 93 | 12/18/2019 | Zhejiang University of Technology | Fire mishaps | Dormitory fire | 0 Fatalities/  0 Injuries/ Facilities  Damage | NetEase News  <https://www.163.com/dy/article/F19FQA9E0514CN9Q.html> |
| 94 | 7/4/2019 | Hainan University | Fire mishaps | Dormitory fire | 0 Fatalities/  0 Injuries/ Facilities  Damage | NetEase News  <https://www.163.com/dy/article/EJD8N4V8053469JX.html> |
| 95 | 10/30/2019 | Unannounced | Fire mishaps | Dormitory fire | 0 Fatalities/  0 Injuries/ Facilities  Damage | China Firefighting  <https://m.thepaper.cn/baijiahao_21029549> |
| 96 | 5/25/2019 | Henan Agricultural University | Fire mishaps | Dormitory fire | 0 Fatalities/  0 Injuries/ Facilities  Damage | Sohu News  <https://www.sohu.com/a/316772786_99999440> |
| 97 | 11/12/2019 | Yantai Institute of Technology | Fire mishaps | Building fires | 0 Fatalities/  0 Injuries/ Facilities  Damage | Baidu News  <https://baijiahao.baidu.com/s?id=1650171941250639876&wfr=spider&for=pc> |
| 98 | 2/27/2019 | Nanjing Tech University | Fire mishaps | Laboratory building fire | 0 Fatalities/  0 Injuries/ Facilities  Damage | NetEase News  <https://www.163.com/dy/article/E91P5UCT0518I06B.html> |
| 99 | 10/29/2019 | Xihua University | Fire mishaps | Canteen fire | 0 Fatalities/  0 Injuries/ Facilities  Damage | Sohu News  <https://www.sohu.com/a/350330968_161795> |
| 100 | 11/28/2019 | Hunan Urban Vocational College | Fire mishaps | Dormitory fire | 1Fatalities/  Facilities  Damage | Shanghai Fire Protection Association Network  <http://www.sh70119.com/news/general/view.php?id=8036> |
| 101 | 3/29/2019 | Beijing Foreign Studies University | Fire mishaps | Dormitory fire | 0 Fatalities/  0 Injuries/ Facilities  Damage | Baidu News  <https://baijiahao.baidu.com/s?id=1629314803344761097&wfr=spider&for=pc> |
| 102 | 3/25/2019 | Central South University of Forestry and Technology | Fire mishaps | Dormitory fire | 0 Fatalities/  0 Injuries/ Facilities  Damage | Weibo News  <https://weibo.com/3549916270/Hn7luqE5K> |
| 103 | 3/14/2019 | Guangxi Construction Vocational Technology College | Fire mishaps | Dormitory fire | 0 Fatalities/  0 Injuries/ Facilities  Damage | NetEase News  <https://www.163.com/dy/article/EAB0CL3T054950HK.html> |
| 104 | 5/6/2019 | Guilin University | Fire mishaps | Dormitory fire | 5 Fatalities/  6 Injuries/ Facilities  Damage | Global Network  <https://baijiahao.baidu.com/s?id=1632789167796574533&wfr=spider&for=pc> |
| 105 | 2/2/2019 | Henan Vocational College of Water Conservancy and Environment | Fire mishaps | Supermarket fire | 0 Fatalities/  0 Injuries/ Facilities  Damage | Baidu News  <https://baijiahao.baidu.com/s?id=1624335805869443696&wfr=spider&for=pc> |
| 106 | 8/28/2019 | Xuchang University | Fire mishaps | Dormitory fire | 0 Fatalities/  0 Injuries/ Facilities  Damage | NetEase News  <https://www.163.com/dy/article/ENQLKO0M05329A0S.html> |
| 107 | 5/25/2019 | Henan Agricultural University | Fire mishaps | Dormitory fire | 0 Fatalities/  0 Injuries/ Facilities  Damage | NetEase News  <https://www.163.com/dy/article/EN9O7JRJ0514MI6D.html> |
| 108 | 3/29/2019 | Jiujiang University | Fire mishaps | Dormitory fire | 0 Fatalities/  0 Injuries/ Facilities  Damage | Sohu News  <https://www.sohu.com/a/306888095_100114174> |
| 109 | 11/11/2019 | Sichuan University of Science & Engineering | Fire mishaps | Dormitory fire | 0 Fatalities/  0 Injuries/ Facilities  Damage | Sohu News  <https://www.sohu.com/a/353094632_99962314> |
| 110 | 6/10/2019 | Mianyang Normal College | Fire mishaps | Dormitory fire | 0 Fatalities/  0 Injuries/ Facilities  Damage | Sohu News  <https://www.sohu.com/a/319608364_202921> |
| 111 | 9/21/2019 | Hainan Normal University | Fire mishaps | Dormitory fire | 0 Fatalities/  0 Injuries/ Facilities  Damage | Sohu News  <https://www.sohu.com/a/430474338_120068650> |
| 112 | 1/8/2019 | Shaoxing University | Fire mishaps | Dormitory fire | 0 Fatalities/  0 Injuries/ Facilities  Damage | Sohu News  <https://www.sohu.com/a/344443474_99903639> |
| 113 | 11/24/2019 | Northwest University of Political Science and Law | Fire mishaps | Academic building fire | 0 Fatalities/  0 Injuries/ Facilities  Damage | Tencent News  <https://www.qq.com/babygohome/?pgv_ref=apub> |
| 114 | 6/10/2019 | Xi’an Jiaotong University | Fire mishaps | Academic building fire | 0 Fatalities/  0 Injuries/ Facilities  Damage | NetEase News  <https://www.163.com/dy/article/EHAS4UOK053109G3.html> |
| 115 | 12/26/2019 | Nanjing University of Posts and Telecommunications | Fire mishaps | Academic building fire | 1 Fatality/  0 Injuries/ Facilities  Damage | Sohu News  <https://www.sohu.com/na/365502276_100293445> |
| 116 | 5/27/2019 | Shanxi Normal University | Fire mishaps | Dormitory fire | 0 Fatalities/  0 Injuries/ Facilities  Damage | know.com  <https://www.zhihu.com/question/327276659> |
| 117 | 9/24/2019 | Xiangtan University | Fire mishaps | Supermarket fire | 0 Fatalities/  0 Injuries/ Facilities  Damage | Sohu Network  <https://www.sohu.com/a/343238371_120348649> |
| 118 | 2/27/2019 | Nanjing Tech University | Laboratory accidents | Laboratory explosion | 0 Fatalities/  0 Injuries/ Facilities  Damage | National Emergency Broadcasting Network  <http://www.cneb.gov.cn/2019/02/27/ARTI1551250292030555.shtml> |
| 119 | 1/1/2019 | Henan Polytechnic University | Unintentional mishaps | fall and die | 1 Fatality | Sohu Network  <https://roll.sohu.com/a/585531799_100277806> |
| 120 | 1/20/2019 | South China University of Technology | Unintentional mishaps | sudden death | 1 Fatality | Sohu Network  <https://www.sohu.com/a/292932386_777213> |
| 121 | 10/27/2019 | Xuzhou Medical University | Unintentional mishaps | sudden death | 1 Fatality | Sohu Network  <https://www.sohu.com/a/350577707_161795> |
| 122 | 5/7/2019 | Tsinghua University | Unintentional mishaps | Fainting | Automatic external defibrillator successfully saves lives | Xinjing News  <https://baijiahao.baidu.com/s?id=1634232646913922585&wfr=spider&for=pc> |
| 123 | 12/26/2019 | Nanjing University of Posts and Telecommunications | Suicide and homicide accidents | Suicide accidents | 1 Fatality | China Youth Network  <https://baijiahao.baidu.com/s?id=1654966641276946993&wfr=spider&for=pc> |
| 124 | 9/2/2019 | Huazhong University of Science and Technology | Suicide and homicide accidents | Suicide accidents | 1 Fatality | Baidu News  <https://baike.baidu.com/item/9%C2%B72%E5%8D%8E%E4%B8%AD%E7%A7%91%E6%8A%80%E5%A4%A7%E5%AD%A6%E7%A1%95%E5%A3%AB%E5%9D%A0%E6%A5%BC%E4%BA%8B%E4%BB%B6/23752945?fr=aladdin> |
| 125 | 4/8/2019 | Wuhan University of Technology | Suicide and homicide accidents | Suicide accidents | 1 Fatality | Sohu Network  <https://www.sohu.com/a/306794850_100229518> |
| 126 | 5/1/2019 | Southern University of Science and Technology | Suicide and homicide accidents | Suicide accidents | 1 Fatality | know.com  <https://www.zhihu.com/question/322534388/answer/676574409> |
| 127 | 12/28/2019 | Shenyang Aerospace University | Suicide and homicide accidents | Suicide accidents | 1 Fatality | NetEase News  <https://m.163.com/dy/article/F1P0GHAS052590Q7.html> |
| 128 | 9/12/2019 | Unannounced | Suicide and homicide accidents | Suicide accidents | 1 Fatality | Sohu Network  <http://news.sohu.com/a/536664019_121124210> |
| 129 | 3/16/2019 | Hunan University of Chinese Medicine | Suicide and homicide accidents | Suicide accidents | 1 Fatality | Baidu News  <http://news.fengone.com/b/20190318/605224.html> |
| 130 | 1/1/2019 | Unannounced | Suicide and homicide accidents | Suicide accidents | 1 Fatality | NetEase News  <https://www.163.com/dy/article/E595UJJ405148UNS.html> |
| 131 | 8/25/2019 | Swan College, Central South University of Forestry and Technology | Suicide and homicide accidents | Homicide accidents | 1 Fatality | NetEase News  <https://www.163.com/dy/article/ENGAS9Q905366EUH.html> |
| 132 | 6/10/2019 | University of Jinan | Suicide and homicide accidents | Suicide accidents | 1 Fatality | Sohu Network  <http://news.sohu.com/a/507932426_120099890> |
| 133 | 9/19/2019 | Anhui Polytechnic University | Suicide and homicide accidents | Homicide accidents | 1 Fatality | China Youth Network  <https://baijiahao.baidu.com/s?id=1682234708048549874&wfr=spider&for=pc> |
| 134 | 9/13/2020 | Shanghai University | Fire mishaps | Pile fires | 0 Fatalities/  0 Injuries/ Facilities  Damage | Sohu News  <https://www.sohu.com/a/418154027_161795> |
| 135 | 9/29/2020 | Chengdu University of Technology | Fire mishaps | Dormitory fire | 0 Fatalities/  0 Injuries/ Facilities  Damage | NetEase News  <https://www.163.com/dy/article/G8F7SOIK05370D2J.html> |
| 136 | 9/29/2020 | Hainan Tropical Ocean University | Fire mishaps | Dormitory fire | 0 Fatalities/  0 Injuries/ Facilities  Damage | Tencent News  <https://new.qq.com/rain/a/20211009A0EKSV00> |
| 137 | 10/13/2020 | Sichuan University | Fire mishaps | Canteen fire | 0 Fatalities/  0 Injuries/ Facilities  Damage | Tencent News  <https://www.qq.com/babygohome/?pgv_ref=apub> |
| 138 | 10/16/2020 | Guangzhou University | Fire mishaps | Dormitory fire | 0 Fatalities/  0 Injuries/ Facilities  Damage | Guangming Network  <https://m.gmw.cn/baijia/2020-10/17/1301682142.html> |
| 139 | 11/19/2020 | Hunan College of Foreign Studies | Fire mishaps | Dormitory fire | 0 Fatalities/  0 Injuries/ Facilities  Damage | NetEase News  <https://www.163.com/dy/article/FRT11CE40545FVKL.html> |
| 140 | 12/21/2020 | Hunan Agricultural University | Fire mishaps | Dormitory fire | 0 Fatalities/  0 Injuries/ Facilities  Damage | Tencent News  <https://new.qq.com/rain/a/20201221A0943U00> |
| 141 | 9/1/2020 | Hunan Agricultural University | Fire mishaps | Dormitory fire | 0 Fatalities/  0 Injuries/ Facilities  Damage | know.com  <https://zhuanlan.zhihu.com/p/339001432> |
| 142 | 6/3/2020 | Hunan Agricultural University, College of Oriental Science and Technology | Fire mishaps | Dormitory fire | 0 Fatalities/  0 Injuries/ Facilities  Damage | Changsha Firefighting  <https://baijiahao.baidu.com/s?id=1686815013694513097&wfr=spider&for=pc> |
| 143 | 9/21/2020 | South China Agricultural University | Fire mishaps | Dormitory fire | 0 Fatalities/  0 Injuries/ Facilities  Damage | Sohu News  <https://www.sohu.com/a/482830146_121123915> |
| 144 | 11/14/2020 | Shantou University | Fire mishaps | Dormitory fire | 0 Fatalities/  0 Injuries/ Facilities  Damage | Sohu News  <https://www.sohu.com/a/431902524_120053840> |
| 145 | 12/31/2020 | Jiangxi Tourism and Commerce Vocational College | Fire mishaps | Dormitory fire | 0 Fatalities/  0 Injuries/ Facilities  Damage | Sina News  <https://k.sina.com.cn/article_2599892125_9af7349d04000y4a7.html> |
| 146 | 11/15/2020 | Weihai Ocean Vocational College | Fire mishaps | Dormitory fire | 0 Fatalities/  0 Injuries/ Facilities  Damage | Global Network  <https://baijiahao.baidu.com/s?id=1683422551028491911&wfr=spider&for=pc> |
| 147 | 12/29/2020 | Unannounced | Fire mishaps | Dormitory fire | 0 Fatalities/  1 Injury/ Facilities  Damage | Sohu News  <http://news.sohu.com/a/442006045_100271702> |
| 148 | 9/19/2020 | Nanjing Agricultural University | Fire mishaps | Dormitory fire | 0 Fatalities/  0 Injuries/ Facilities  Damage | Sohu News  <https://www.sohu.com/a/421207401_120209938> |
| 149 | 10/29/2020 | Wuchang Institute of Technology | Fire mishaps | Dormitory fire | 0 Fatalities/  0 Injuries/ Facilities  Damage | Hongshan Firefighting  <https://www.meipian.cn/388e3zcq> |
| 150 | 1/14/2020 | Huazhong Agricultural University | Fire mishaps | Academic building fire | 0 Fatalities/  0 Injuries/ Facilities  Damage | Hongshan Firefighting  <https://www.meipian.cn/388e3zcq> |
| 151 | 9/8/2020 | Wuhan Institute of Technology | Fire mishaps | Dormitory fire | 0 Fatalities/  0 Injuries/ Facilities  Damage | Hongshan Firefighting  <https://www.meipian.cn/388e3zcq> |
| 152 | 9/18/2020 | WUHAN CITY POLYTECHNIC | Fire mishaps | Dormitory fire | 0 Fatalities/  0 Injuries/ Facilities  Damage | Hongshan Firefighting  <https://www.meipian.cn/388e3zcq> |
| 153 | 9/22/2020 | Unannounced | Fire mishaps | Academic building fire | 0 Fatalities/  0 Injuries/ Facilities  Damage | Hongshan Firefighting  <https://www.meipian.cn/388e3zcq> |
| 154 | 11/24/2020 | Unannounced | Fire mishaps | Dormitory fire | 0 Fatalities/  0 Injuries/ Facilities  Damage | Sohu News  <https://www.sohu.com/a/434828859_205720> |
| 155 | 12/1/2020 | Huabei Health College | Fire mishaps | Dormitory fire | 0 Fatalities/  0 Injuries/ Facilities  Damage | NetEase News  <https://www.163.com/dy/article/FSRGFH7H05444NVR.html> |
| 156 | 10/14/2020 | Guangxi Arts University | Fire mishaps | Academic building fire | 0 Fatalities/  0 Injuries/ Facilities  Damage | Asset Management Office of Beijing University of Posts and Telecommunications  <https://zcc.bupt.edu.cn/info/1093/1323.htm> |
| 157 | 11/9/2020 | Minzu University of China | Fire mishaps | Dormitory fire | 0 Fatalities/  0 Injuries/ Facilities  Damage | know.com  <https://www.zhihu.com/question/429458725> |
| 158 | 11/10/2020 | Capital Normal University | Fire mishaps | Gymnasium fire | 0 Fatalities/  0 Injuries/ Facilities  Damage | Baidu News  <https://baike.baidu.com/item/%E9%A6%96%E9%83%BD%E5%B8%88%E8%8C%83%E5%A4%A7%E5%AD%A6%E4%BD%93%E8%82%B2%E9%A6%86%E8%B5%B7%E7%81%AB%E4%BA%8B%E4%BB%B6/16776271?fr=aladdin> |
| 159 | 10/22/2020 | Harbin Sport University | Fire mishaps | Dormitory fire | 0 Fatalities/  0 Injuries/ Facilities  Damage | NetEase News  <https://www.163.com/dy/article/FPQ6VLRN0531O7QG.html> |
| 160 | 8/5/2020 | Nanjing Tech University | Fire mishaps | Academic building fire | 0 Fatalities/  0 Injuries/ Facilities  Damage | Xinjing News  <https://baijiahao.baidu.com/s?id=1626592323070337126&wfr=spider&for=pc> |
| 161 | 12/21/2020 | Southwest Jiaotong University | Laboratory accidents | Chemical combustion | 0 Fatalities/  0 Injuries/ Facilities  Damage | School of Materials Science and Engineering, Southwest Jiaotong University  <https://clxy.swjtu.edu.cn/info/1110/14586.htm> |
| 162 | 11/6/2020 | Xi’an Eurasia University | Unintentional mishaps | Fallen building | 2 Injuries | Global Network  <https://baijiahao.baidu.com/s?id=1682687815896569731&wfr=spider&for=pc> |
| 163 | 11/7/2020 | Henan University of Engineering | Unintentional mishaps | Fallen building | 1 Fatality | Redstar News  <https://baijiahao.baidu.com/s?id=1684421486470454009&wfr=spider&for=pc> |
| 164 | 12/30/2020 | Dalian University of Technology | Unintentional mishaps | Car Accident | 1 Fatality | Sohu News  <https://www.sohu.com/a/618454151_121295251> |
| 165 | 12/3/2020 | Sichuan Tourism University | Unintentional mishaps | Falling | 1 Fatality | Tencent News  <https://www.qq.com/babygohome/?pgv_ref=apub> |
| 166 | 9/27/2020 | Xuzhou Medical University | Unintentional mishaps | Fallen building | 1 Fatality | China Network  <https://baijiahao.baidu.com/s?id=1679320439386260742&wfr=spider&for=pc> |
| 167 | 11/19/2020 | Guangdong Polytechnic | Unintentional mishaps | Fainting | 1 Fatality | Baidu News  <https://baijiahao.baidu.com/s?id=1684383491329095351&wfr=spider&for=pc> |
| 168 | 9/21/2020 | Nanning University | Unintentional mishaps | Food poisoning | Unannounced | Weibo News  <https://weibo.com/ttarticle/p/show?id=2309354552299929272431> |
| 169 | 11/25/2020 | Harbin Institute of Technology | Unintentional mishaps | sudden death | 1 Fatality | NetEase News  <https://www.163.com/dy/article/FSF1AOG405444WMT.html> |
| 170 | 9/14/2020 | China University of Geosciences Beijing | Unintentional mishaps | sudden death | 1 Fatality | NetEase News  <https://m.163.com/dy/article/GQQ8D1N60514EV7Q.html> |
| 171 | 11/11/2020 | Beihang University | Unintentional mishaps | sudden death | 1 Fatality | NetEase News  <https://www.163.com/dy/article/FRQSDR5L0536NCEV.html> |
| 172 | 9/12/2020 | Nanjing Normal University | Unintentional mishaps | sudden death | 1 Fatality | China Newsweek  <https://baijiahao.baidu.com/s?id=1678617213242286095&wfr=spider&for=pc> |
| 173 | 10/2/2020 | Guangdong University of Technology | Unintentional mishaps | sudden death | 1 Fatality | Sohu News  <https://www.sohu.com/a/423246964_531924> |
| 174 | 9/15/2020 | Northeastern University | Unintentional mishaps | sudden death | 1 Fatality | Xinjing News  <https://baijiahao.baidu.com/s?id=1678801945086567883&wfr=spider&for=pc> |
| 175 | 6/10/2020 | Chengdu University of Technology | Suicide and homicide accidents | Homicide accidents | 2 Injuries | Baidu News  <https://baijiahao.baidu.com/s?id=1669170673243083801&wfr=spider&for=pc> |
| 176 | 10/12/2020 | Jiangsu University | Suicide and homicide accidents | Suicide accidents | 1 Fatality | Tencent News  <https://new.qq.com/rain/a/20201017A060WR00> |
| 177 | 12/28/2020 | Guangdong University of Petrochemical Technology | Suicide and homicide accidents | Homicide accidents | 3 Injuries | Baidu News  <https://baike.baidu.com/item/12%C2%B728%E5%B9%BF%E4%B8%9C%E7%9F%B3%E6%B2%B9%E5%8C%96%E5%B7%A5%E5%AD%A6%E9%99%A2%E6%B3%BC%E7%A1%AB%E9%85%B8%E6%A1%88/55668244?fr=aladdin> |
| 178 | 11/25/2020 | Sichuan Agricultural University | Suicide and homicide accidents | Suicide accidents | 1 Fatality | Baidu News  <https://tieba.baidu.com/p/7116403912> |
| 179 | 10/13/2020 | Dalian University of Technology | Suicide and homicide accidents | Suicide accidents | 1 Fatality | Baidu News  <https://baike.baidu.com/item/10%C2%B713%E5%A4%A7%E8%BF%9E%E7%90%86%E5%B7%A5%E5%A4%A7%E5%AD%A6%E7%A0%94%E7%A9%B6%E7%94%9F%E8%87%AA%E6%9D%80%E4%BA%8B%E4%BB%B6/54050831?fr=aladdin> |
| 180 | 6/6/2020 | North University of China | Suicide and homicide accidents | Suicide accidents | 1 Fatality | Global Network  <https://baijiahao.baidu.com/s?id=1669020143403728120&wfr=spider&for=pc> |
| 181 | 9/19/2020 | Nanjing University | Suicide and homicide accidents | Suicide accidents | 1 Fatality | Sohu News  <https://www.sohu.com/a/477222295_121124021> |
| 182 | 12/4/2020 | Nanjing University | Suicide and homicide accidents | Suicide accidents | 1 Fatality | Baidu News  <https://www.cn-healthcare.com/articlewm/20210715/content-1243276.html> |
| 183 | 5/3/2020 | Communication University of China | Suicide and homicide accidents | Suicide accidents | 1 Fatality | China Youth Network  <https://baijiahao.baidu.com/s?id=1666451759510809416&wfr=spider&for=pc> |
| 184 | 7/6/2020 | Hunan University | Suicide and homicide accidents | Suicide accidents | 1 Fatality | Sohu News  <https://roll.sohu.com/a/629591642_121252400> |
| 185 | 11/2/2020 | Hunan Normal University | Suicide and homicide accidents | Suicide accidents | 1 Fatality | Baidu News  <https://baijiahao.baidu.com/s?id=1682722518204975243&wfr=spider&for=pc> |
| 186 | 8/18/2020 | Nanjing University of Aeronautics and Astronautics | Suicide and homicide accidents | Suicide accidents | 1 Fatality | know.com  <https://zhuanlan.zhihu.com/p/266191399> |
| 187 | 8/31/2020 | Nanjing University of Aeronautics and Astronautics | Suicide and homicide accidents | Suicide accidents | 1 Fatality | know.com  <https://zhuanlan.zhihu.com/p/266191399> |
| 188 | 9/7/2020 | Zhejiang University | Suicide and homicide accidents | Suicide accidents | 1 Fatality | know.com  <https://zhuanlan.zhihu.com/p/266191399> |
| 189 | 9/3/2020 | Shanghai Jiao Tong University | Suicide and homicide accidents | Suicide accidents | 1 Fatality | Sohu News  <https://www.sohu.com/a/423372082_531924> |
| 190 | 9/19/2020 | Shanxi Jinzhong Institute of Technology | Suicide and homicide accidents | Suicide accidents | 1 Fatality | Weibo News  <https://weibo.com/3630020045/JlM8zf7MP> |
| 191 | 10/×/2020 | Sichuan University | Suicide and homicide accidents | Suicide accidents | 1 Fatality | know.com  <https://zhuanlan.zhihu.com/p/266191399> |
| 192 | 10/10/2020 | Nanjing Audit University | Suicide and homicide accidents | Suicide accidents | 1 Fatality | know.com  <https://zhuanlan.zhihu.com/p/266191399> |
| 193 | 12/15/2020 | Beijing Jiaotong University | Suicide and homicide accidents | Suicide accidents | 1 Fatality | Baidu News  <https://baijiahao.baidu.com/s?id=1686681941151925581&wfr=spider&for=pc> |
| 194 | 10/26/2020 | Zhengzhou Business University | Suicide and homicide accidents | Suicide accidents | 1 Fatality | Baidu News  <https://baijiahao.baidu.com/s?id=1682790719352130444&wfr=spider&for=pc> |
| 195 | 10/5/2020 | Zhengzhou University | Suicide and homicide accidents | Suicide accidents | 1 Fatality | Baidu News  <https://baijiahao.baidu.com/s?id=1684605635525264185&wfr=spider&for=pc> |
| 196 | 10/9/2020 | West China Hospital， Sichuan University | Suicide and homicide accidents | Suicide accidents | 1 Fatality | know.com  <https://www.zhihu.com/question/424816745> |
| 197 | 10/19/2020 | Chengdu University of Technology | Suicide and homicide accidents | Suicide accidents | 1 Fatality | Baidu News  <https://baijiahao.baidu.com/s?id=1681957471963610169&wfr=spider&for=pc> |
| 198 | 10/22/2020 | Beijing Normal University | Suicide and homicide accidents | Suicide accidents | 1 Fatality | know.com  <https://www.zhihu.com/question/422230277/answer/1486278270> |
| 199 | 10/24/2020 | Zhongnan University of Economics and Law | Suicide and homicide accidents | Suicide accidents | 1 Fatality | know.com  <https://www.zhihu.com/question/427107892> |
| 200 | 10/27/2020 | Beijing Institute of Technology | Suicide and homicide accidents | Suicide accidents | 1 Fatality | NetEase News  <https://www.163.com/dy/article/FQGOQ450054829NU.html> |
| 201 | 11/2/2020 | Hunan Normal University Business School | Suicide and homicide accidents | Suicide accidents | 1 Fatality | Baidu News  <https://baijiahao.baidu.com/s?id=1682792109620387516&wfr=spider&for=pc> |
| 202 | 11/5/2020 | Hefei University of Technology | Suicide and homicide accidents | Suicide accidents | 1 Fatality | Baidu News  <https://baijiahao.baidu.com/s?id=1685161712767230329&wfr=spider&for=pc> |
| 203 | 11/16/2020 | Shanghai University | Suicide and homicide accidents | Suicide accidents | 1 Fatality | NetEase News  <https://www.163.com/dy/article/FSAEARR50545BCNA.html> |
| 204 | 11/20/2020 | Sun Yat-sen University | Suicide and homicide accidents | Suicide accidents | 1 Fatality | know.com  <https://www.zhihu.com/question/431587532> |
| 205 | 11/28/2020 | Sun Yat-sen University | Suicide and homicide accidents | Suicide accidents | 1 Fatality | know.com  <https://www.zhihu.com/question/432370385/answer/1599969390> |
| 206 | 11/30/2020 | Hainan University | Suicide and homicide accidents | Suicide accidents | 1 Fatality | know.com  <https://www.zhihu.com/question/358578842> |
| 207 | 10/10/2020 | Lanzhou Petrochemical University of Vocational Technology | Suicide and homicide accidents | Suicide accidents | 2 Fatalities | Baidu News  <https://baijiahao.baidu.com/s?id=1681302321781722777&wfr=spider&for=pc> |
| 208 | 10/6/2021 | South China Agricultural University | Fire mishaps | Dormitory fire | 0 Fatalities/  0 Injuries/ Facilities  Damage | Sohu News  <https://www.sohu.com/a/583340210_121123756> |
| 209 | 8/3/2021 | Heilongjiang University of Chinese Medicine | Fire mishaps | Dormitory fire | 0 Fatalities/  0 Injuries/ Facilities  Damage | NetEase News  <https://www.163.com/dy/article/GLUF8UU90552IB1C.html> |
| 210 | 4/27/2021 | Hangzhou Dianzi University | Fire mishaps | Dormitory fire | 0 Fatalities/  0 Injuries/ Facilities  Damage | Tencent News  <https://new.qq.com/rain/a/ZJC2021042800253906> |
| 211 | 4/4/2021 | Guangxi University | Fire mishaps | Academic building fire | 0 Fatalities/  0 Injuries/ Facilities  Damage | Sohu News  <https://www.sohu.com/a/637840002_121106875> |
| 212 | 12/13/2021 | Guangdong University of Technology | Fire mishaps | Canteen fire | 0 Fatalities/  0 Injuries/ Facilities  Damage | Baidu News  <https://m.thepaper.cn/baijiahao_15859453> |
| 213 | 10/19/2021 | Jilin Jianzhu University | Fire mishaps | Canteen fire | 0 Fatalities/  0 Injuries/ Facilities  Damage | Sina News  <http://k.sina.com.cn/article_1558725752_m5ce84478053014ycl.html> |
| 214 | 12/24/2021 | Fujian Normal University | Fire mishaps | Dormitory fire | 0 Fatalities/  0 Injuries/ Facilities  Damage | China Youth Network  <https://baijiahao.baidu.com/s?id=1620727229664181180&wfr=spider&for=pc> |
| 215 | 10/25/2021 | Shandong University of Science and Technology | Fire mishaps | Canteen fire | 0 Fatalities/  0 Injuries/ Facilities  Damage | NetEase News  <https://m.163.com/dy/article/GN6IMR110514ET7F.html> |
| 216 | 3/14/2021 | Southeast University | Fire mishaps | Dormitory fire | 0 Fatalities/  0 Injuries/ Facilities  Damage | Sohu News  <https://www.sohu.com/a/500154615_121124306> |
| 217 | 5/15/2021 | Baise University | Fire mishaps | School bus on fire | 0 Fatalities/  0 Injuries/ Facilities  Damage | Baidu News  <https://baijiahao.baidu.com/s?id=1699976629193134417&wfr=spider&for=pc> |
| 218 | 7/13/2021 | Southern University of Science and Technology | Fire mishaps | Fire in the laboratory building | 0 Fatalities/  1 Injury/ Facilities  Damage | Baidu News  <https://m.thepaper.cn/baijiahao_15062997> |
| 219 | 9/21/2021 | Hainan University | Fire mishaps | Dormitory fire | 0 Fatalities/  0 Injuries/ Facilities  Damage | China News Network  <https://www.chinanews.com/edu/2013/09-22/5306523.shtml> |
| 220 | 1/8/2021 | Sichuan Normal University | Fire mishaps | Dormitory fire | 0 Fatalities/  0 Injuries/ Facilities  Damage | Sichuan Normal University Security Office  <https://bwc.sicnu.edu.cn/_wx/_wx_home_news_i.aspx?iid=637457247247315248> |
| 221 | 5/20/2021 | Chengdu University of Technology | Fire mishaps | Dormitory fire | 0 Fatalities/  0 Injuries/ Facilities  Damage | North Youth Network  <https://t.ynet.cn/baijia/30837293.html> |
| 222 | 8/2/2021 | Jinan University | Fire mishaps | Dormitory fire | 0 Fatalities/  0 Injuries/ Facilities  Damage | Baidu News  <https://baijiahao.baidu.com/s?id=1707825189098105215&wfr=spider&for=pc> |
| 223 | 5/8/2021 | Beijing Normal University | Fire mishaps | Dormitory fire | 0 Fatalities/  0 Injuries/ Facilities  Damage | Baidu News  <https://m.thepaper.cn/baijiahao_21029549> |
| 224 | 9/28/2021 | Guizhou University | Fire mishaps | Academic building fire | 0 Fatalities/  0 Injuries/ Facilities  Damage | Sina News  <http://k.sina.com.cn/article_7517400647_m1c0126e4705901hbw5.html> |
| 225 | 5/2/2021 | Guizhou Medical University | Fire mishaps | Canteen fire | 0 Fatalities/  0 Injuries/ Facilities  Damage | Baidu News  <https://baijiahao.baidu.com/s?id=1699093284153041533&wfr=spider&for=pc> |
| 226 | 10/25/2021 | South China Agricultural University | Fire mishaps | Dormitory fire | 0 Fatalities/  0 Injuries/ Facilities  Damage | Baidu News  <https://www.thepaper.cn/newsDetail_forward_15095353> |
| 227 | 10/9/2021 | Unannounced | Fire mishaps | Dormitory fire | 0 Fatalities/  1 Injury/ Facilities  Damage | Sohu News  <https://www.sohu.com/a/492803076_121123813> |
| 228 | 11/17/2021 | Communication University of China, Nanjing | Fire mishaps | Dormitory fire | 0 Fatalities/  0 Injuries/ Facilities  Damage | Baidu News  <https://baijiahao.baidu.com/s?id=1716754629304989385&wfr=spider&for=pc> |
| 229 | 3/31/2021 | Institute of Chemistry, Chinese Academy of Sciences | Laboratory accidents | Laboratory explosion | 1 Fatality/  0 Injuries/ Facilities  Damage | Baidu News  <https://baike.baidu.com/item/3%C2%B731%E4%B8%AD%E7%A7%91%E9%99%A2%E5%8C%96%E5%AD%A6%E6%89%80%E7%88%86%E7%82%B8%E4%BA%8B%E6%95%85/56562416> |
| 230 | 10/24/2021 | Nanjing University of Aeronautics and Astronautics | Laboratory accidents | Laboratory explosion | 2 Fatalities/  9 Injuries/ Facilities  Damage | Xinjing News  <https://baijiahao.baidu.com/s?id=1714514283031787864&wfr=spider&for=pc> |
| 231 | 7/27/2021 | Sun Yat-sen University | Laboratory accidents | Laboratory explosion | 0 Fatalities/  1 Injury/ Facilities  Damage | Baidu News  <https://baijiahao.baidu.com/s?id=1706593604072914286&wfr=spider&for=pc> |
| 232 | 7/13/2021 | Southern University of Science and Technology | Laboratory accidents | Chemical combustion | 0 Fatalities/  1 Injury/ Facilities  Damage | Baidu News  <https://m.thepaper.cn/baijiahao_15062997> |
| 233 | 4/22/2021 | Sichuan University | Unintentional mishaps | sudden death | 1 Fatality | Baidu News  <https://baijiahao.baidu.com/s?id=1698276272353410346&wfr=spider&for=pc> |
| 234 | 9/23/2021 | Hubei University of Technology | Unintentional mishaps | Fall and die | 1 Fatality | North Youth Network  <https://t.ynet.cn/baijia/31480294.html> |
| 235 | 11/23/2021 | Liaoning Technical University | Unintentional mishaps | sudden death | 1 Fatality | Baidu News  <https://baijiahao.baidu.com/s?id=1718357473315387814&wfr=spider&for=pc> |
| 236 | 12/21/2021 | Linyi University | Unintentional mishaps | sudden death | 1 Fatality | Baidu News  <https://baijiahao.baidu.com/s?id=1721179390287320730&wfr=spider&for=pc> |
| 237 | 4/29/2021 | Baise University | Unintentional mishaps | sudden death | 1 Fatality | Baidu News  <https://baike.baidu.com/item/4%C2%B729%E7%99%BE%E8%89%B2%E5%AD%A6%E9%99%A2%E5%AD%A6%E7%94%9F%E7%AF%AE%E7%90%83%E5%9C%BA%E6%99%95%E5%80%92%E4%BA%8B%E4%BB%B6/56948293?fr=aladdin> |
| 238 | 12/15/2021 | Xi'an MINGDE INSTITUTE OF TECHNOLOGY | Unintentional mishaps | sudden death | 1 Fatality | Baidu News  <https://baijiahao.baidu.com/s?id=1719748193501312048&wfr=spider&for=pc> |
| 239 | 11/22/2021 | Sun Yat-sen University | Unintentional mishaps | Cardiac arrest | 1 person was successfully treated | Baidu News  <https://baijiahao.baidu.com/s?id=1742007961292242785&wfr=spider&for=pc> |
| 240 | 9/25/2021 | Shandong Traffic Technician College | Unintentional mishaps | sudden death | 1 Fatality | Baidu News  <https://baijiahao.baidu.com/s?id=1712034800998875863&wfr=spider&for=pc> |
| 241 | 4/21/2021 | Central South University | Suicide and homicide accidents | Suicide accidents | 1 Fatality | Baidu News  <https://baike.baidu.com/item/4%C2%B721%E4%B8%AD%E5%8D%97%E5%A4%A7%E5%AD%A6%E7%A1%95%E5%A3%AB%E7%94%9F%E5%9D%A0%E6%A5%BC%E4%BA%8B%E4%BB%B6/56802763?fr=aladdin> |
| 242 | 4/8/2021 | Nanjing University | Suicide and homicide accidents | Suicide accidents | 1 Fatality | Baidu News  <https://www.cn-healthcare.com/articlewm/20210429/content-1215354.html> |
| 243 | 3/13/2021 | Nanjing University | Suicide and homicide accidents | Suicide accidents | 1 Fatality | Baidu News  <https://www.cn-healthcare.com/articlewm/20210429/content-1215354.html> |
| 244 | 10/22/2021 | Yan’an University | Suicide and homicide accidents | Suicide accidents | 1 Fatality | NetEase News  <https://www.163.com/dy/article/H5TRKUN00552ZLKT.html> |
| 245 | 9/15/2021 | Shenyang Ligong University | Suicide and homicide accidents | Homicide accidents | 1 Fatality | Baidu News  <https://baijiahao.baidu.com/s?id=1736943559339961223&wfr=spider&for=pc> |
| 246 | 6/14/2021 | Wuhan University | Suicide and homicide accidents | Suicide accidents | 1 Fatality | NetEase News  <https://m.163.com/dy/article/GJFHQJD20545BCNA.html> |
| 247 | 5/13/2021 | Fujian Agriculture and Forestry University | Suicide and homicide accidents | Suicide accidents | 1 Fatality | Baidu News  <https://baijiahao.baidu.com/s?id=1699992349478123358&wfr=spider&for=pc> |
| 248 | 3/23/2021 | Zhengzhou College of Finance and Economics | Suicide and homicide accidents | Suicide accidents | 1 Fatality | Baidu News  <https://baijiahao.baidu.com/s?id=1695187823659396492&wfr=spider&for=pc> |
